# Crystallinity‐Enhanced CO_2_ Adsorption by Sodium Poly(Heptazine Imide) Frameworks

**DOI:** 10.1002/cssc.202500775

**Published:** 2025-07-12

**Authors:** Pedro Ouro, Álvaro Cuevas, Johannes Liessem, Dariusz Mitoraj, Radim Beranek, Eva Díaz, Salvador Ordóñez, Ildefonso Marin‐Montesinos, Daniel Pereira, Mariana Sardo, Igor Krivtsov, Luís Mafra, Marina Ilkaeva

**Affiliations:** ^1^ Department of Chemistry CICECO – Aveiro Institute of Materials University of Aveiro Campus Universitário de Santiago 3810‐193 Aveiro Portugal; ^2^ Department of Chemical and Environmental Engineering University of Oviedo 33006 Oviedo Spain; ^3^ Institute of Electrochemistry Ulm University Albert‐Einstein‐Allee 47 89081 Ulm Germany

**Keywords:** adsorption, carbon dioxide capture, carbon nitrides, ionothermal syntheses, poly(heptazine) imides

## Abstract

This work presents sodium poly(heptazine imide) (NaPHI)‐based materials, synthesized in a NaCl medium, as highly effective platforms for CO_2_ capture. High crystallinity—an often‐overlooked aspect in PHI frameworks—is identified as a key factor governing CO_2_ adsorption capacity in microporous structures. Thermogravimetric analysis and manometric studies reveal a CO_2_ uptake of ≈3.8 mmol g^−1^, at 1 bar and 25 °C, surpassing most reported PHI‐based adsorbents under similar conditions. Exchanging Na^+^ with K^+^ or Rb^+^ preserves CO_2_ adsorption performance, whereas Cs^+^ incorporation induces structural distortion, greatly reducing CO_2_ adsorption capacity in PHI. These materials exhibit excellent cyclic stability (20 cycles) without degradation and CO_2_ adsorption capacity loss. Notably, at flue gas‐relevant temperature (100 °C), NaPHI attains a CO_2_ capacity of 2.1 mmol g^−1^, doubling the performance of benchmark Zeolite 13X (1.1 mmol g^−1^). Ideal Adsorbed Solution Theory confirms remarkable CO_2_/N_2_ selectivity (≈3.8 mmol g^−1^ vs typical N_2_ adsorption of 0.3 mmol g^−1^), a critical property for postcombustion CO_2_ capture. These findings position PHI‐based materials as a disruptive platform for CO_2_ adsorption, offering 1) straightforward synthesis from readily available precursors, 2) promising scalability, and 3) outstanding performance.

## Graphical abstract

High CO_2_ capacity (≈3.8 mmol g^−1^, 25 °C, 1 bar, and pure CO_2_) and selectivity (Ideal Adsorbed Solution Theory selectivity ≈234, for a CO_2_ feed of 15%) are achieved applying sodium poly(heptazine imide) adsorbent. The adsorbent retains high CO_2_ capacity of 2.1 mmol g^−1^ even at flue gas‐relevant temperature (100 °C).

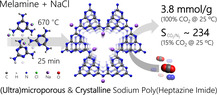

## 1 Introduction

Several classes of solid adsorbents have been reported as effective candidates for CO_2_ adsorption, such as metal‐organic frameworks (MOFs),^[^ ^1^, ^2^, ^3^ ^]^ covalent‐organic frameworks (COFs),^[^ ^4^ ^]^ Activated Carbons (ACs),^[^ ^5^, ^6^, ^7^ ^]^ biochars,^[^ ^8^, ^9^, ^10^ ^]^ resins,^[^ ^11^ ^]^ and zeolites.^[^ ^12^, ^13^ ^]^ The latter are already widely used in industry for gas stream purification from CO_2_.^[^ ^14^, ^15^ ^]^ Zeolites present an anionic Si/Al oxide framework counterbalanced by alkali or alkaline‐earth cations, which possess high polarizability and a high quadrupolar moment. These cations in zeolites were found to polarize CO_2_ molecules and then reposition within the zeolites’ structural cavities. This process allows CO_2_ to enter the micropores, leading to high selectivity in CO_2_ adsorption from gas mixtures (e.g., CO_2_/N_2_, CO_2_/CH_4_).^[^ ^16^, ^17^ ^]^ However, zeolites also present weaknesses, namely, 1) H_2_O and CO_2_ competition for the same active sites,^[^ ^18^ ^]^ 2) susceptibility to hydrolysis at high temperatures,^[^ ^19^ ^]^ and 3) cost‐intensive regeneration.^[^ ^20^ ^]^ Therefore, the search for highly selective CO_2_ sorbents operating via similar mechanisms as zeolites, such as Zeolite 13X (the zeolite of reference in CO_2_ capture), is of paramount importance.

Poly(heptazine imides) (PHIs),^[^ ^21^, ^22^, ^23^, ^24^, ^25^, ^26^, ^27^, ^28^, ^29^ ^]^ ionic members of a broader family of polymeric carbon nitrides (PCNs),^[^ ^30^ ^]^ emerge as promising candidates. Like zeolites, they feature negatively charged frameworks counterbalanced by cations introduced during ionothermal synthesis.^[^ ^31^ ^]^ Although PCNs as well as PHIs possess bridge and terminal amino groups in their structures, their contribution to CO_2_ capture is expected to be relatively low as these groups are involved in H‐bonding between the heptazine layers rendering them unable to chemically bind CO_2_.^[^ ^32^ ^]^ However, unlike the PCN, mostly amorphous and presenting no defined porous structure, leading to its low CO_2_ adsorption capacity,^[^ ^33^ ^]^ PHI materials can be obtained in a more crystalline form. Indeed, Dontsova et al. pioneered the synthesis of a series of crystalline PHI materials obtained via the molten‐salt technique using eutectic KCl/LiCl and individual Li, Na, K, Rb, and Cs halides as the reaction medium.^[^ ^34^, ^35^ ^]^ Later, Lotsch et al. developed a method for highly crystalline KPHI synthesis, and its structure could be resolved by PXRD methods.^[^ ^21^ ^]^ The structural and computational studies demonstrated that such polymeric heptazine frameworks must possess structural pores of subnanometer size,^[^ ^21^, ^23^, ^27^ ^]^ therefore making it a promising material for small‐molecule adsorption. The computational studies suggest that even He could be captured in such polarized environment of the alkali metal PHI cavity.^[^ ^36^ ^]^ Recent studies determined that fragmented, poorly crystalline PCN and PHI materials with high surface areas (≈334 m^2^ g^−1^, which is high for this class of materials) achieved CO_2_ adsorption capacities of ≈2.2 mmol g^−1^, in flue gas conditions (at 15% CO_2_, 25 °C), however, their performance was limited by structural disorder.^[^ ^27^ ^]^ This highlights that a high surface area alone is not sufficient for the material to guarantee efficient CO_2_ capture.

In this work, we demonstrate that controlling the crystallinity of PHI materials addresses this limitation by optimizing structure and functionality (CO_2_ binding sites), leading to a better performance in CO_2_ capture. Enhanced in‐plane order in crystalline NaPHI not only preserves micropore accessibility but also stabilizes alkali metal cations within the framework, turning them from hindrances into active contributors. The resulting ordered structure provides well‐defined binding sites and efficient CO_2_ diffusion pathways, enabling the crystalline NaPHI to achieve a high CO_2_ uptake of 3.3 mmol g^−1^ (15% CO_2_) and 3.8 mmol g^−1^ (100% CO_2_), at 25 °C and 1 bar.

## 2 Results and Discussion

### 2.1 Characterization of NaPHI‐Based Materials

#### 2.1.1 Structural Properties

**Figure 1:**
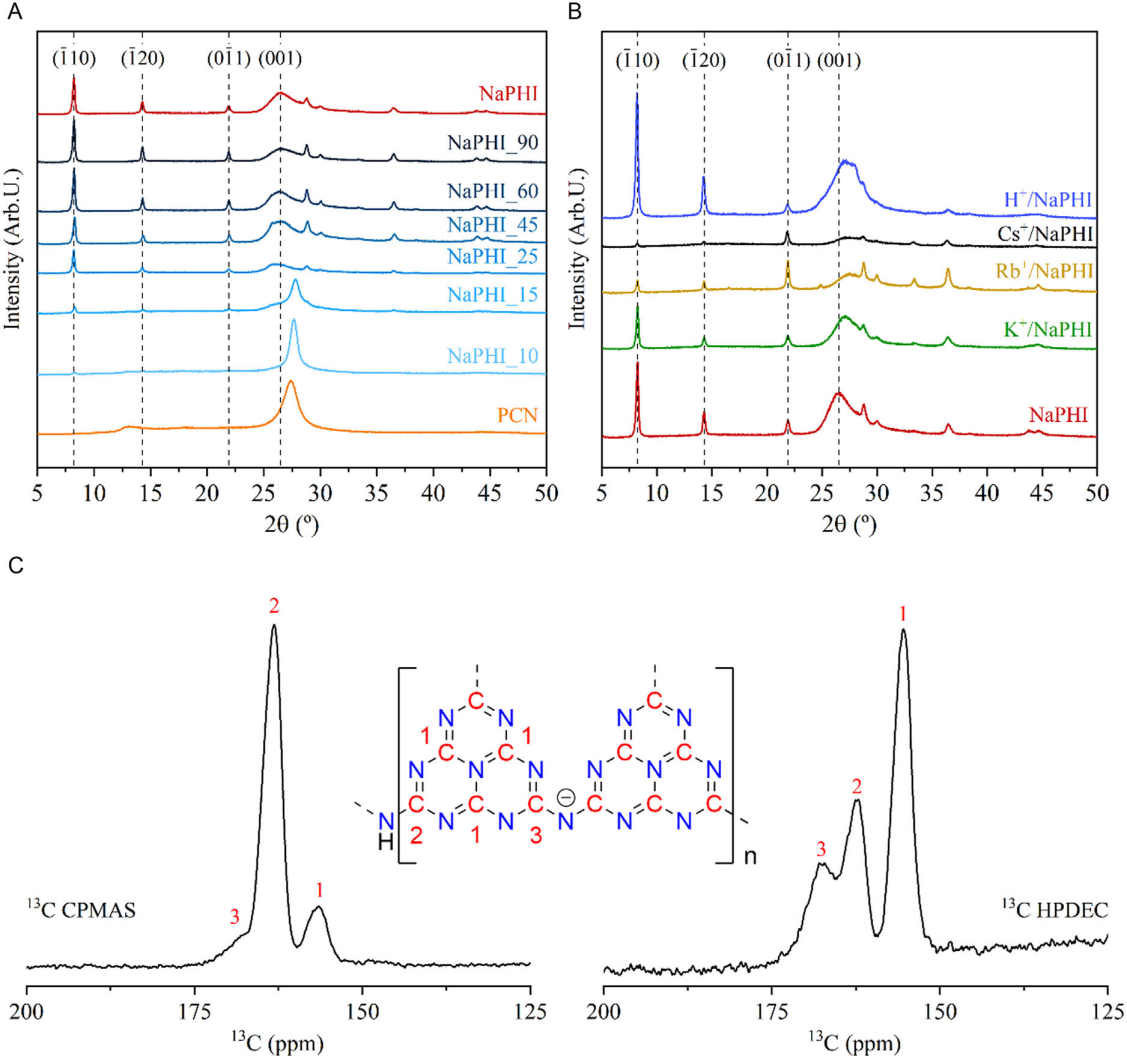
A) PXRD diffractograms comparing **PCN** with **NaPHI_T** (synthesized at 650 °C) samples (where *T* is the dwell time in minutes), and the parent **NaPHI** sample (synthesized at 670 °C, for 25 min); B) PXRD diffractograms of **NaPHI** and its ion‐exchanged analogues; and C) ^13^C solid‐state nuclear magnetic resonance (ssNMR) spectra of **NaPHI**; Field strength of 9.4 T, and a spinning frequency of 11111 Hz.

PXRD data (**Figure** **1**A, S1, Supporting Information) show that the NaPHI phase^[^ ^21^, ^31^, ^34^ ^]^ is gradually developed from an amorphous PCN‐like polymer into a crystalline material upon increasing time of the thermal treatment. The PXRD reflections of **NaPHI** sample can be indexed to a triclinic unit cell, according to the work of Lotsch et al., which reports the elucidation of isostructural potassium PHI.^[^ ^21^ ^]^ The broad reflection near 26.5° (001) is attributed to the stacking of multiple PHI layers.^[^ ^21^ ^]^ The corrugation of the PHI sheets does not allow for an ordered out‐of‐plane stacking, hence a broad distribution of interlayer distances is observed. The high relative intensity of the peak at 8.2° ($\bar{1}10$), with respect to others indicates high intralayer order of the synthesized material. This contrasts with other PHIs prepared from KCl‐ or KSCN‐containing melts^[^ ^21^, ^28^, ^34^ ^]^ and from CaCl_2_ molten salt.^[^ ^27^ ^]^ Noteworthy, the peak corresponding to the $\left(\bar{1}20\right)$ reflection is observed at 14° for **NaPHI**, while for KPHI it manifests itself at 15°. According to the simulations made by Lotsch et al., this may correspond to the eclipsed conformation of the PHI sheets in NaPHI^[^ ^21^ ^]^ (Figure 1A). It is hypothesized that this configuration results in the formation of channels by superposition of structural inter‐heptazine voids making them a potential site for CO_2_ adsorption. Indeed, the preliminary CO_2_ adsorption studies clearly show that the increased crystallinity is conducive of enhanced CO_2_ uptake reaching the optimal performance by the **NaPHI** sample (Figure S2 and S3, Supporting Information).

With the objective of assessing the effect of different metal cations on the parent PHI's physicochemical properties and CO_2_ adsorption performance, we carried out the ion‐exchange of the parent **NaPHI** yielding **K** ^**+**^ **/NaPHI**, **Rb** ^**+**^ **/NaPHI, Cs** ^**+**^ **/NaPHI**, and **H** ^**+**^ **/NaPHI**. The efficiency of the ion‐exchange was assessed through ICP‐MS (**Table** **1**), and the results showed that for all analogues, most Na^+^ ions were successfully replaced by the used cations or protons.

**Table 1:** ICP‐MS analysis results of ion‐exchanged **NaPHI**, where X is the cation (K^+^, Rb^+^, Cs^+^, or H^+^) introduced into the PHI.

Samples	Na^+^ [mmol g^−1^]	X [mmol g^−1^]	Na^+^ [mg g^−1^]	X [mg g^−1^]
**NaPHI**	3.17	0.00	73.00	0.00
**K** ^**+**^ **/NaPHI**	0.07	2.30	1.70	89.70
**Rb** ^**+**^ **/NaPHI**	0.06	3.67	1.37	314.00
**Cs** ^**+**^ **/NaPHI**	0.30	1.29	6.92	171.20
**H** ^**+**^ **/NaPHI**	0.03	0.00	0.58	0.00

Noteworthy, the crystalline structure of the parent **NaPHI** is preserved when Na^+^ is exchanged by H^+^, K^+^, and Rb^+^ as the same series of reflections can be clearly identified on the PXRD patterns (Figure 1B). This crystalline preservation, regardless of the cation size, indicates the long‐range structural order and robustness of the **NaPHI's** polymer backbone.^[^ ^34^ ^]^ Nonetheless, the intensity of the peak at 8.2° ($\bar{1}10$) decreases for the ion‐exchanged samples in the order H^+^ > Na^+^ > K^+^ > Rb^+^. Apparently, larger cations contribute to a greater degree of disorder in the ($\bar{1}10$) direction. In fact, the insertion of Cs^+^ into the PHI framework results in almost total disappearance of the maximum at 8.2°, indicating that the material might be suffering amorphization due to irregular Cs^+^ packing.^[^ ^31^ ^]^ Proton incorporation also causes a shift of the interlayer stacking peak, from 26.5° to ≈27.1°, indicating the shortening of the interlayer distances.

^13^C cross‐polarization (CP), magic‐angle spinning (MAS), and ^13^C high‐power decoupling (HPDEC) ssNMR spectra of the **NaPHI** sample (Figure 1C) provided information on the local environment of the structural carbons, revealing three resonances: C1 (*δ* = 155.5), C2 (*δ* = 162.4), and C3 (*δ* = 167.6 ppm).^[^ ^27^ ^]^ These resonances correspond to carbons in —C=N— bonds within heptazines, carbons near protonated —NH— bridges and residual uncondensed —NH_2_ functions, and carbons adjacent to deprotonated —C—N^−^—C, respectively.^[^ ^37^ ^]^ The resonances ascribed to other expected structural carbons, such as those adjacent to or within residual surface nitrile (—N=C=N^−^) groups could also be present in the material. However, their resonances are not observed due to their low concentration and inherent low sensitivity of ^13^C NMR experiments. FTIR spectroscopy is in accordance with this assignment, as it reveals the typical set of bands for PHI (Figure S4, Supporting Information) of the prepared **NaPHI** material, especially the absorption bands at 990 and 1065 cm^−1^ corresponding to the symmetric and asymmetric vibrations of —C—N^−^—C— bonds of —C—N(metal)—C— groups^[^ ^38^ ^]^ and the low‐intensity peak at about 2200 cm^−1^ attributed to vibrations of the residual nitrile groups.

According to these results, **NaPHI's** structure differs significantly from CaPHI's reported by Burrow et al.,^[^ ^27^ ^]^ another PHI‐based material used for CO_2_ adsorption. While **NaPHI** exhibits high crystallinity, calcium incorporation in CaPHI likely results in a more disordered polymer with an increased number of protonated C—N— end‐functions. These structural differences between **NaPHI** and CaPHI may lead to significant differences in their ability to capture CO_2_.^[^ ^27^ ^]^ The results retrieved from additional characterization techniques, such as elemental analysis (EA), thermogravimetric analysis coupled with mass spectrometry (TGA‐MS), scanning electron microscopy (SEM), scanning and transmission electron microscopy (STEM), and energy dispersive X‐Ray spectroscopy (EDX) are discussed in Supporting Information (Table S1–S4, Figure S5–S9, Supporting Information).

#### 2.1.2 Textural Properties

**Figure 2:**
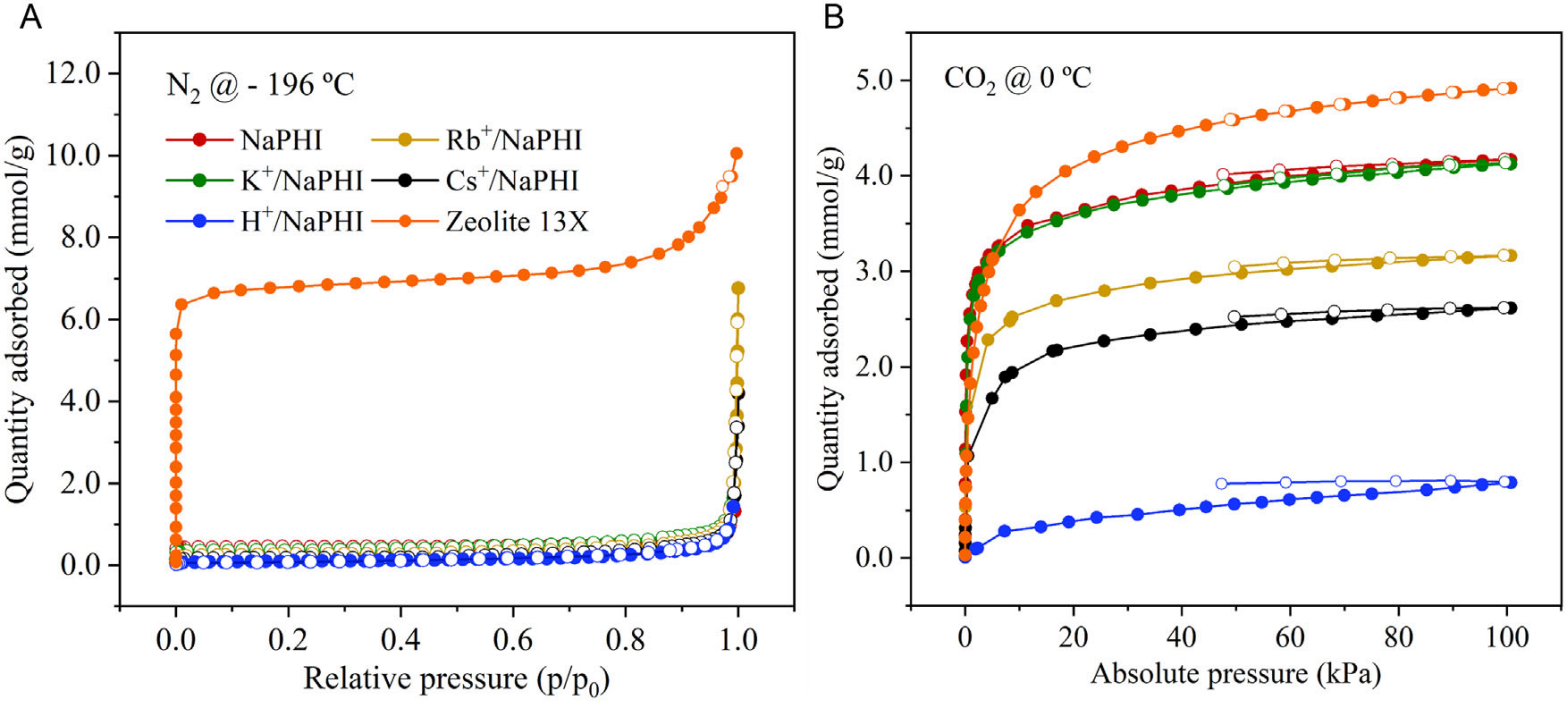
Adsorption‐desorption isotherms of A) N_2_ at −196 °C and B) CO_2_ at 0 °C of **NaPHI**, its ion‐exchanged analogues, and **Zeolite 13X**.

N_2_ adsorption–desorption isotherms at −196 °C (**Figure** **2**A, S10, Table S5, Supporting Information) revealed that all prepared PHI‐based materials have low BET surface area, with the highest one belonging to **K** ^**+**^ **/NaPHI** at 27 m^2^ g^−1^ (**Table** **2**). All PHI's isotherms are of type III, according to IUPAC classification, which is typical of non‐porous solids and usually reported for crystalline PHIs.^[^ ^34^, ^39^ ^]^ Also, the N_2_ adsorption–desorption profile of PHIs is dissimilar to benchmark CO_2_ sorbent **Zeolite 13X**, which is of type I, typical of microporous materials (Figure 2A). Nonetheless, the observed low adsorption of N_2_ at −196 °C does not necessarily mean the absence of micropores suitable for CO_2_ capture. Nitrogen has a larger kinetic diameter than CO_2_, and its stick‐like geometry may impede it from entering the micropores of <0.7 nm size.^[^ ^40^ ^]^ Therefore, the variations of the BET surface area values observed for the series of NaPHI‐based materials can be attributed to a different assembly of the PHI sheets within the material and do not reflect changes in their microporous structures (Table 2, S5, Supporting Information). Therefore, CO_2_ isotherms at 0 °C were registered, in order to determine the size of the **NaPHI** micropores (Figure 2B). A steep CO_2_ uptake is observed, at low pressures, which is indicative of high affinity of the material to the adsorbate and could be due to the presence of micropores in **NaPHI** and its ion‐exchanged analogues. It is worth noting that the desorption branch of the CO_2_ isotherm of the PHI samples does not coincide with the adsorption one at the experimental equilibration conditions, which might be due to strong interactions of the adsorbate with PHI or to its small pore size that hinders the CO_2_ desorption (Figure 2B).

**Table 2:** Data retrieved from the N_2_ at −196 °C and CO_2_ at 0 °C adsorption isotherms of **NaPHI**, its ion‐exchanged analogues and **Zeolite 13X**.

Sample	*S*_BET_ a) [m^2^ g^−1^]	*V*_Total_ a) [cm^3^ g^−1^]	CO_2_ [0 °C] [mmol g^−1^]	*V*_micro_ b) [cm^3^ g^−1^]
**NaPHI**	25	0.04	4.17	0.21
**K** ^**+**^ **/NaPHI**	27	0.05	4.14	0.21
**Rb** ^**+**^ **/NaPHI**	16	0.06	3.17	0.17
**Cs** ^**+**^ **/NaPHI**	11	0.05	2.61	0.14
**H** ^**+**^ **/NaPHI**	8	0.05	0.79	0.10
**Zeolite 13X**	604	0.33	4.91	0.27

a) Data retrieved from N_2_ adsorption isotherms at −196 °C.

b) Data retrieved from CO_2_ adsorption isotherms, at 0 °C. *V*_micro_ —total micropore volume calculated from the CO_2_ adsorption isotherms at 0 °C using the Dubinin–Stoeckli model.

The application of the GCMC model to **Zeolite 13X's** N_2_ adsorption–desorption isotherm showed an average pore width of 1.1 nm (Figure S11, Supporting Information), which is in good agreement with the literature data.^[^ ^41^, ^42^ ^]^ To determine the pore size distribution (PSD) of **NaPHI** from its respective CO_2_ adsorption–desorption isotherms, at 0 °C, the Dubinin–Stoeckli (DS) model was applied^[^ ^43^ ^]^ (Figure S12, Supporting Information). The results reveal the presence of pores with an average pore width of 0.7 nm. Hence, **NaPHI** presents pores in the range of (super)micropores and (ultra)micropores,^[^ ^44^ ^]^ which is an excellent attribute for CO_2_ capture.

### 2.2 The Cation Effect in CO_2_ Capture Performance

#### 2.2.1 Thermogravimetric and Manometric CO_2_ Adsorption

**Figure 3:**
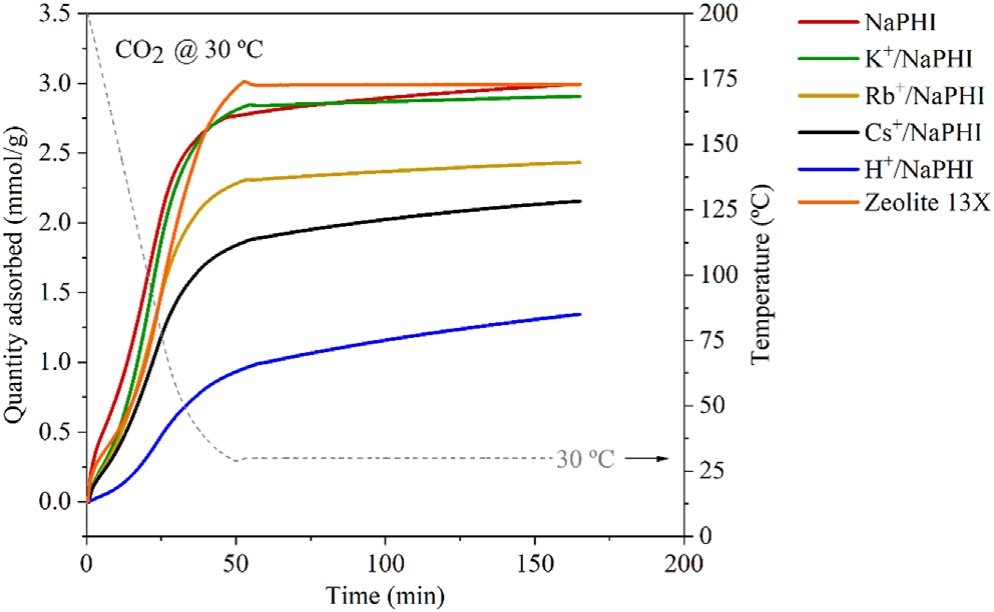
TGA CO_2_ adsorption measurements for **NaPHI** and its ion‐exchange analogues.

According to the TGA adsorption measurements, **NaPHI** reaches a high CO_2_ adsorption capacity of ≈3.0 mmol g^−1^ (30 °C, 1 bar CO_2_) (**Figure** **3**). Such performance is comparable with the benchmark **Zeolite 13X** that, under similar conditions, also adsorbs 3.0 mmol g^−1^ of CO_2_. The ion‐exchanged **NaPHI** analogues also present satisfactory CO_2_ adsorption capacities, even though their capacity seems to decrease with the increase in the size of the incorporated cation (Figure 3). Interestingly, the exchange of Na^+^ for H^+^ led to the lowest registered CO_2_ adsorption capacity, which could be explained by the reduced polarizability of CO_2_ by protons as compared to the alkali metal cations (Figure 3, **Table** **3**). Importantly, all PHI‐based materials also show lower adsorption capacity towards N_2_ than to CO_2_ indicating its higher affinity to carbon dioxide (Figure S13, Supporting Information).

**Table 3:** CO_2_ adsorption capacities of **NaPHI** and its ion‐exchanged analogues. a) CO_2_ adsorption capacity normalized to the total mass of the sample. b) CO_2_ adsorption capacity normalized to the mass of the dehydrated at 200 °C sample. c) CO_2_ adsorption capacity normalized to the mass of the dehydrated at 200 °C sample from which the mass of metal cations was excluded.

Sample	Thermogravimetric data [30 °C]			Manometric data [25 °C]	
	CO_2_ [mmol g^−1^]			CO_2_ [mmol g^−1^]	
	(a)	(b)	(c)	(b)	(c)
**NaPHI**	3.02 ± 0.03	3.81 ± 0.02	4.11 ± 0.06	3.80	4.10
**K** ^**+**^ **/NaPHI**	2.88 ± 0.03	3.52 ± 0.02	3.88 ± 0.02	3.70	4.07
**Rb** ^**+**^ **/NaPHI**	2.45 ± 0.01	2.87 ± 0.02	4.20 ± 0.04	2.70	3.94
**Cs** ^**+**^ **/NaPHI**	2.04 ± 0.13	2.41 ± 0.15	2.92 ± 0.17	2.30	2.75
**H** ^**+**^ **/NaPHI**	1.23 ± 0.13	1.59 ± 0.17	1.59 ± 0.17	0.82	0.82

**Figure 4:**
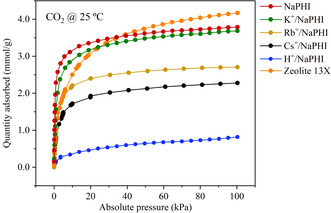
Manometric CO_2_ adsorption study, at 25 °C, of **NaPHI** and its ion‐exchanged analogues.

The CO_2_ adsorption capacity of the ion‐exchanged **NaPHI** samples was also evaluated by manometric measurements to assess the adsorption performance of the sorbents, at different partial pressures of CO_2_ (**Figure** **4**). As expected, all isotherms show high affinity of **NaPHI** and its ion‐exchanged materials for CO_2_, at 25 °C, even at low partial pressures. This is indicative of a microporous PHI framework, which is crucial for CO_2_ adsorption, especially in dilute gaseous streams.^[^ ^45^ ^]^ The CO_2_ uptake by **Zeolite 13X** at low partial pressures is not as steep as for most PHI‐based (Na, K, Rb, and Cs) sorbents. This higher affinity of the latter for CO_2_ could be due to a narrower pore size that favors CO_2_ adsorption. In contrast, the H^+^‐exchanged analogue presented a drastic decrease in adsorption capacity owing to the reduced basicity of the PHI framework. The CO_2_ adsorption capacities estimated by the manometric measurements (at 25 °C) are in good agreement with those obtained via the TGA method (at 30 °C) (Table 3).

N_2_ adsorption isotherms, under the same conditions, present a linear trend with increasing N_2_ partial pressure, thus evidencing a low affinity of all the PHI materials as well as **Zeolite 13X** towards this gas (Figure S13B, Supporting Information). N_2_ adsorption assessed by manometric measurements afforded lower values of adsorption capacity than that carried out by TGA. The principal factor that can explain this discrepancy is the kinetic impediment that nitrogen molecules encounter upon incorporation into the PHI framework at the relatively low adsorption temperature of 25 °C. The kinetics of N_2_ adsorption can be favored by the applied temperature program during the TGA measurements, where the initial adsorption temperature is 200 °C, gradually decreasing to 30 °C.

Interestingly, the normalization of the CO_2_ adsorption capacities by the mass of the dehydrated material and excluding the mass of the metal cations reveals that the parent **NaPHI** polymeric framework is barely affected by the exchange of Na^+^ for K^+^ or Rb^+^ affording values of 4.11, 3.88, and 4.20 mmol g^−1^ (PHI), respectively (Table 3). However, the size of the cation may affect the kinetics of the CO_2_ adsorption process, which seems to be slower with larger cations, as it is evident by the slope of the CO_2_ uptake on the TGA and manometric measurements (Figure 3 and 4).

Contrary to the observations reported by Burrow et al.,^[^ ^27^ ^]^ the exchange of alkali metal cation in the PHI structure for H^+^ leads to a drastic decrease of CO_2_ uptake. The hypothesis that the reduction of adsorption capacity can be caused by distortion of the PHI structure induced by H^+^ incorporation was ruled out, according to the PXRD data. (Figure 1B). Therefore, it is hypothesized that, even though protons possess the smallest ionic radius (steric hinderance is reduced), the protonation of the PHI framework decreases its basicity, affording a weaker direct interaction with CO_2_, leading to the observed reduced CO_2_ adsorption capacity. This agrees with observations made by Walton et al., who argued that the increased framework‐basicity of zeolites results in their stronger interactions with CO_2_.^[^ ^46^ ^]^

#### 2.2.2 CO_2_/N_2_ Adsorption Selectivity

The IAST was applied to the CO_2_ and N_2_ isotherms acquired at 25 °C and fitted with the dual‐site Langmuir and Henry models (Figures S14, S15, Tables S6–S8, Supporting Information**)**.

**Figure 5:**
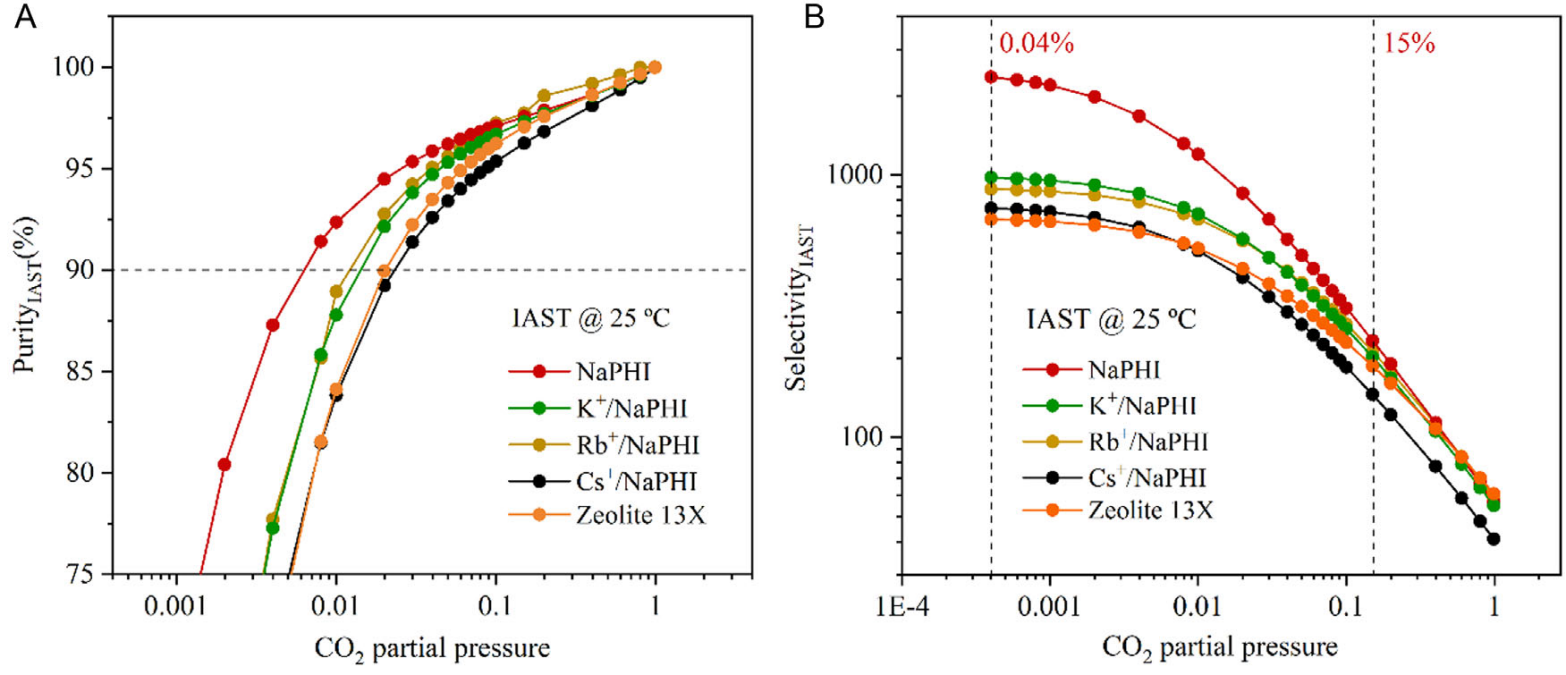
Data retrieved from employing IAST equations to CO_2_ and N_2_ isotherms, recorded at 25 °C, of **NaPHI** and its ion‐exchanged analogues, and **Zeolite 13X**: A) IAST purity and B) IAST selectivity.

The IAST selectivity (**Figure** **5**A) is significantly higher at lower partial pressures of CO_2_, for all assessed PHI‐based materials than it is for **Zeolite 13X**, converging into similar values as the total pressure approaches 1 bar. The most selective PHI‐based material is **NaPHI**, presenting an IAST selectivity of ≈2500, for a CO_2_ feed of 0.04%, and ≈234, for a CO_2_ feed of 15%, which is significantly more selective than **Zeolite 13X**, especially at low CO_2_ partial pressures (IAST selectivity of ≈676, for a CO_2_ feed of 0.04%, and ≈187, for a CO_2_ feed of 15%). To corroborate these results, Henry's law constants were retrieved from the linear region of the CO_2_ isotherms at 25 °C (Figure S15, Table S7, Supporting Information). Then, the apparent selectivity of the PHI‐based materials was calculated based on Henry's law constants for both CO_2_ and N_2_ isotherms, at 25 °C, and compared to the IAST‐selectivity values obtained, showing only slight differences (Table S8, Supporting Information).

As for IAST purity (Figure 5A), the results show that all PHI‐based materials are well within the targets set by regulatory committees of over 90% purity of the captured CO_2_.^[^ ^47^ ^]^ All assessed PHI‐based materials with incorporated alkali cations, apart from **Cs** ^**+**^ **/NaPHI**, can achieve these targets at pressures lower than 0.01 bar (or CO_2_ feeds <1%), which shows that this polymeric framework is extremely well‐suited for application in dilute gas streams.

#### 2.2.3 Temperature Effect on CO_2_ Adsorption and Adsorbent Recyclability

**Figure 6:**
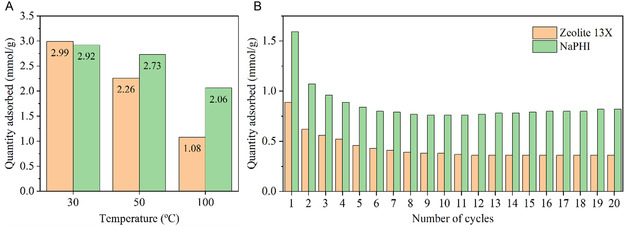
CO_2_ adsorption capacity from TGA tests for **NaPHI** and **Zeolite 13X:** A) adsorption at different temperatures and B) adsorption–desorption cycles at an adsorption temperature of 100 °C (for (A), pretreatment was performed at 200 °C, for 2 h, under N_2_ atmosphere; for (B), the pretreatment was 200 °C for cycle 1, then from cycle 2 to cycle 20, it was 100 °C, 20 min for regeneration and 10 min for CO_2_ adsorption); CO_2_ flow rate for all measurements was 40 mL min^−1^, at 1 bar).

The **NaPHI** material and the benchmark **Zeolite 13X** were chosen for the study of the temperature effect on the CO_2_ adsorption capacity, which was assessed at 30, 50, 100, 150, and 200 °C by TGA. The choice of the benchmark adsorbent is justified by our hypothesis that the CO_2_ adsorption mechanisms of **Zeolite 13X** and our adsorbent are similar, as they both present Na^+^ in their micropores for the charge compensation of their anionic frameworks.^[^ ^48^, ^49^ ^]^ The CO_2_ adsorption capacity results (**Figure** **6**A and Table S9, Supporting Information) show that **NaPHI** exhibits comparable CO_2_ adsorption capacity to **Zeolite 13X** (≈3.0 mmol g^−1^) at 30 °C, 100% CO_2_. However, at higher temperatures, **NaPHI** outperforms **Zeolite 13X (**Figure S16A, Table S9, Supporting Information**)**. Particularly, we would like to highlight a significantly improved performance of **NaPHI** over **Zeolite 13X**, at 100 °C, where **NaPHI** achieves nearly double of the CO_2_ adsorption capacity than **Zeolite 13X** (2.1 vs. 1.1 mmol g^−1^). Given that postcombustion flue gas streams typically range in temperature from 75 to 120 °C, the results at 100 °C showcase a significant advantage of using **NaPHI** over **Zeolite 13X**, making it an excellent choice for flue‐gas carbon capture. Even when comparing **NaPHI** with other adsorbents evaluated under postcombustion capture conditions (namely, in the flue gas temperature range), it performs exceptionally well against other physisorbents and even presents an adsorption capacity which is comparable to that of the chemisorbents (Table S10, Supporting Information).

Apart from reaching high adsorption capacity, the adsorbent should be easily regenerated by temperature and pressure swing (Figure 6B, S16, Supporting Information). Therefore, to assess recyclability, both **NaPHI** and **Zeolite 13X** were subjected to 20 adsorption–desorption cycles, at two different adsorption temperatures: 30 °C (Figure S16B, Supporting Information) and 100 °C (Figure 6B).

**NaPHI** afforded excellent results after 20 cycles, maintaining a consistent 1.8 mmol g^−1^ of CO_2_ adsorption capacity from cycle 12 to cycle 20 for the adsorption temperature of 30 °C (Figure S16B, Supporting Information). As expected, a significant decrease in the adsorption capacities, from cycle 1 to cycle 2, occurs due to the decrease in the desorption temperature from 200 to 100 °C applied in between cycles for the remaining cycles of the measurement. At this temperature, **Zeolite 13X** maintained a consistent 2.2 mmol g^−1^ of CO_2_ adsorption capacity from cycle 12 to cycle 20. The performance of the two materials changes dramatically upon increasing the adsorption temperature to 100 °C. A significant reduction in CO_2_ adsorption capacity was observed for both **NaPHI** and **Zeolite 13X**. This time, **NaPHI** presented a consistent 0.8 mmol g^−1^ of CO_2_ adsorption capacity while **Zeolite 13X** showed a 0.4 mmol g^−1^ throughout 20 cycles of adsorption. These results showed that, despite **Zeolite 13X**'s superior CO_2_ adsorption capacity in cyclic adsorption studies, at 30 °C, **NaPHI** presents higher CO_2_ adsorption capacity under elevated adsorption temperatures, which more closely resemble realistic conditions for CO_2_ adsorption from flue gas. Structural stability of **NaPHI** was also assessed by performing 20 adsorption–desorption cycles while maintaining the same desorption temperature (200 °C) in between adsorption–desorption cycles. The resulting data showed that **NaPHI's** CO_2_ adsorption capacity does not change significantly with each cycle, which signifies that **NaPHI** is stable in the established conditions and its crystalline structure is retained after this experiment (Figures S17 and S18, Supporting Information).

## 3 Conclusions

We have reported a microporous **NaPHI** framework with improved crystallinity and enhanced CO_2_ adsorption capacity reaching values of 3.8 mmol g^−1^ at 1 bar CO_2_ and 25 °C, which is superior to that of other types of polymeric carbon nitride‐based sorbents. The formation of the PHI framework in NaCl medium renders the material high in‐plane order and results in the eclipsed stacking of the poly(heptazine) sheets, thus producing microporous channels where CO_2_ can be trapped. It has been observed that ion‐exchange of Na^+^ for other alkali metal cations such as K^+^ and Rb^+^ does not significantly affect the ability of the framework to capture CO_2_, while the incorporation of larger Cs^+^ distorts the polymer structure leading to the loss of its adsorption capacity. It is also noteworthy that for our crystalline PHI materials, the adsorption capacity is greatly reduced when alkali metal cations in their structure are replaced by H^+^, while the contrary was previously observed for disordered amine‐rich PHIs. We have demonstrated that crystallinity, rather than surface area alone, governs the efficiency of CO_2_ adsorption in PHI materials by ensuring structural integrity and strategic cation placement—key factors for advancing carbon capture materials. In addition, we also demonstrated that the synthesized **NaPHI** adsorbent not only has comparable adsorption capacity to the commercial benchmark **Zeolite 13X,** at near room temperature conditions, but it even outperforms it in terms of selectivity in binary CO_2_/N_2_ gas mixtures and in terms of capacity at elevated temperatures of adsorption, close to the flue‐gas conditions. Furthermore, cyclic adsorption and recyclability studies confirmed not only an exceptional stability of **NaPHI** but also its ability to regenerate to full initial adsorption capacity by a simple temperature swing. These results highlight the immense potential of **NaPHI** for CO_2_ adsorption, especially in flue gas adsorption conditions or from very diluted gas streams.

## 4 Experimental Section

The NaPHI adsorbent was synthesized by a modified ionothermal method reported by Dontsova et al.^[^ ^34^ ^]^ For the details on materials synthesis, characterization, and CO_2_ and N_2_ adsorption measurements see Supporting Information.

## Conflict of Interest

The authors declare no conflict of interest.

## Supporting information

Supplementary Material

## Data Availability

The data that support the findings of this study are available from the corresponding author upon reasonable request.

## References

[1] C. A. Trickett , A. Helal , B. A. Al‐Maythalony , Z. H. Yamani , K. E. Cordova , O. M. Yaghi , Nat. Rev. Mater. 2017, 2, 17045.

[2] Z. Sun , Y. Liao , S. Zhao , X. Zhang , Q. Liu , X. Shi , J. Mater. Chem. A 2022, 4, 5174.

[3] W. L. Li , Q. Shuai , J. Yu , Small 2024, 20, 2402783. 10.1002/smll.202402783 39115100

[4] H. Li , A. Dilipkumar , S. Abubakar , D. Zhao , Chem. Soc. Rev. 2023, 52, 6294. 37591809 10.1039/d2cs00465h

[5] S. Acevedo , L. Giraldo , J. C. Moreno‐Piraján , ACS Omega 2020, 5, 10423. 32426599 10.1021/acsomega.0c00342 PMC7226889

[6] H. Sugiyama , Y. Hattori , Chem. Phys. Lett. 2020, 758, 137909.

[7] M. S. Tam , M. J. Antal , Ind. Eng. Chem. Res. 1999, 38, 4268.

[8] A. D. Igalavithana , S. W. Choi , P. D. Dissanayake , J. Shang , C. H. Wang , X. Yang , S. Kim , D. C. W. Tsang , K. B. Lee , Y. S. Ok , J. Hazard. Mater. 2020, 391, 121147. 32145924 10.1016/j.jhazmat.2019.121147

[9] M. V. Nguyen , B. K. Lee , Process Saf. Environ. Prot. 2016, 104, 490.

[10] M. A. O. Lourenço , T. Frade , M. Bordonhos , M. Castellino , M. L. Pinto , S. Bocchini , Chem. Eng. J. 2023, 470, 144005.

[11] M. Parvazinia , S. Garcia , M. Maroto‐Valer , J. Chem. Eng. 2018, 331, 335.

[12] Z. Tao , Y. Tian , W. Wu , Z. Liu , W. Fu , C.‐W. Kung , J. Shang , npj Mater. Sustainability 2024, 2, 20.

[13] F. Raganati , F. Miccio , P. Ammendola , Energy Fuels 2021, 19, 12845.

[14] F. Mwacharo , S. Bhandari , A. Othman , A.‐R. Rautio , Biogas Drying and Purification Methods; can be found under 2020, https://www.theseus.fi/handle/10024/356234 (accessed: June 2025).

[15] A. Sayari , Y. Belmabkhout , R. Serna‐Guerrero , Chem. Eng. J. 2011, 171, 760.

[16] J. Shang , G. Li , R. Singh , P. Xiao , J. Z. Liu , P. A. Webley , J. Phys. Chem. C 2013, 117, 12841.

[17] Q. L. Liu , A. Mace , Z. Bacsik , J. L. Sun , A. Laaksonen , N. Hedin , Chem. Commun. 2010, 46, 4502. 10.1039/c000900h 20428579

[18] J. A. Mason , T. M. McDonald , T. H. Bae , J. E. Bachman , K. Sumida , J. J. Dutton , S. S. Kaye , J. R. Long , J. Am. Chem. Soc. 2015, 137, 4787. 25844924 10.1021/jacs.5b00838

[19] C. J. Heard , L. Grajciar , C. M. Rice , S. M. Pugh , P. Nachtigall , S. E. Ashbrook , R. E. Morris , Nat. Commun. 2019, 10, 4690. 31619677 10.1038/s41467-019-12752-y PMC6795794

[20] B. Wu , X. Zhang , Y. Xu , D. Bao , S. Zhang , J. Cleaner Prod. 2015, 101, 251.

[21] H. Schlomberg , J. Kröger , G. Savasci , M. W. Terban , S. Bette , I. Moudrakovski , V. Duppel , F. Podjaski , R. Siegel , J. Senker , R. E. Dinnebier , C. Ochsenfeld , B. V. Lotsch , Chem. Mater. 2019, 31, 7478. 31582875 10.1021/acs.chemmater.9b02199 PMC6768190

[22] A. Savateev , M. Antonietti , ChemCatChem 2019, 11, 6166.

[23] F. Podjaski , B. V. Lotsch , Adv. Energy Mater. 2021, 11, 2003049.

[24] A. Savateev , D. Dontsova , B. Kurpil , M. Antonietti , J. Catal. 2017, 350, 203.

[25] F. K. Kessler , Y. Zheng , D. Schwarz , C. Merschjann , W. Schnick , X. Wang , M. J. Bojdys , Nat. Rev. Mater. 2017, 2, 17030.

[26] G. F. S. R. Rocha , M. A. R. da Silva , A. Rogolino , G. A. A. Diab , L. F. G. Noleto , M. Antonietti , I. F. Teixeira , Chem. Soc. Rev. 2023, 52, 4878. 37409655 10.1039/d2cs00806h

[27] J. N. Burrow , R. A. Ciufo , L. A. Smith , Y. Wang , D. C. Calabro , G. Henkelman , C. B. Mullins , ACS Nano 2022, 16, 5393. 35358382 10.1021/acsnano.1c08912

[28] J. N. Burrow , J. P. Pender , J. V. Guerrera , B. R. Wygant , J. E. Eichler , D. C. Calabro , C. B. Mullins , C. B. Mullins , ACS Appl. Nano Mater. 2020, 3, 5965.

[29] C. M. Pelicano , M. Antonietti , Angew. Chem. Int. Ed. 2024, 63, e202406290. 10.1002/anie.202406290 38687031

[30] X. Wang , K. Maeda , A. Thomas , K. Takanabe , G. Xin , J. M. Carlsson , K. Domen , M. Antonietti , Nat. Mater. 2009, 8, 76. 18997776 10.1038/nmat2317

[31] A. Savateev , S. Pronkin , M. G. Willinger , M. Antonietti , D. Dontsova , Chem. Asian J. 2017, 12, 1517. 28199049 10.1002/asia.201700209

[32] A. Schwarzer , T. Saplinova , E. Kroke , Coord. Chem. Rev. 2013, 257, 2032.

[33] S. N. Talapaneni , G. Singh , I. Y. Kim , K. AlBahily , A. H. Al‐Muhtaseb , A. S. Karakoti , E. Tavakkoli , A. Vinu , Adv. Mater. 2020, 32, 1904635. 10.1002/adma.201904635 31608512

[34] Z. Chen , A. Savateev , S. Pronkin , V. Papaefthimiou , C. Wolff , M. G. Willinger , E. Willinger , D. Neher , M. Antonietti , D. Dontsova , Adv. Mater. 2017, 29, 1700555. 10.1002/adma.201700555 28632318

[35] A. Savateev , S. Pronkin , J. D. Epping , M. G. Willinger , C. Wolff , D. Neher , M. Antonietti , D. Dontsova , ChemCatChem 2017, 9, 167.

[36] S. K. Sahoo , J. Heske , S. Azadi , Z. Zhang , N. V. V. Tarakina , M. Oschatz , R. Z. Khaliullin , M. Antonietti , T. D. Kühne , Sci. Rep. 2020, 10, 5832. 32242048 10.1038/s41598-020-62638-z PMC7118168

[37] J. Kröger , A. Jiménez‐Solano , G. Savasci , P. Rovó , I. Moudrakovski , K. Küster , H. Schlomberg , H. A. Vignolo‐González , V. Duppel , L. Grunenberg , C. B. Dayan , M. Sitti , F. Podjaski , C. Ochsenfeld , B. V. Lotsch , Adv. Energy Mater. 2021, 11, 2003016.

[38] D. C. Bradley , M. H. Gitlitz , J. Chem. Soc. 1969, 980.

[39] Q. Deng , H. Li , W. Hu , W. Hou , Angew. Chem., Int. Ed. 2023, 62, e202314213. 10.1002/anie.202314213 37794843

[40] D. Lozano‐Castelló , D. Cazorla‐Amorós , A. Linares‐Solano , Carbon 2004, 42, 1233.

[41] C. Chen , D. W. Park , W. S. Ahn , Appl. Surf. Sci. 2014, 292, 63.

[42] Y. Yu , L. Zheng , J. Wang , J. Air Waste Manage. 2012, 62, 1227. 10.1080/10962247.2012.702186 23155869

[43] M. M. Dubinin , H. F. Stoeckli , J. Colloid Interface Sci. 1980, 75, 34.

[44] M. Thommes , K. Kaneko , A. V. Neimark , J. P. Olivier , F. Rodriguez‐Reinoso , J. Rouquerol , K. S. W. Sing , Pure Appl. Chem. 2015, 87, 1051.

[45] M. Oschatz , M. Antonietti , Energy Environ. Sci. 2018, 11, 57.

[46] K. S. Walton , M. B. Abney , M. D. LeVan , Microporous Mesoporous Mater. 2006, 91, 78.

[47] Quality guidelines for energy system studies CO_2_ Impurity Design Parameters; can be found under 2019, https://www.netl.doe.gov (accessed: June 2025).

[48] D. P. Bezerra , F. W. M. D. Silva , P. A. S. D. Moura , A. G. S. Sousa , R. S. Vieira , E. Rodriguez‐Castellon , D. C. S. Azevedo , Appl. Surf. Sci. 2014, 314, 314.

[49] A. Caravella , G. Prenesti , S. De Luca , M. Turano , F. Testa , R. Girimonte , Separations 2023, 10, 558.

