# Supplementary material for: Crystallinity‐Enhanced CO2 Adsorption by Sodium Poly(Heptazine Imide) Frameworks
Source: ChemSusChem. 2025 Jul 12;18(16):e202500775. doi: 10.1002/cssc.202500775 (PMC12330333; doi:10.1002/cssc.202500775)
Supplement: Supplementary file 1 — Supplementary Material [file CSSC-18-e202500775-s001.pdf]

SUPPORTING INFORMATION

Crystallinity-Enhanced CO_2 Adsorption by Sodium Poly(Heptazine Imide) Frameworks

Pedro Ouro[a,b], Álvaro Cuevas[b], Johannes Liessem[c], Dariusz Mitoraj[c], Radim Beranek[c], Eva Díaz[b], Salvador Ordóñez[b], Ildefonso Marin-Montesinos[a], Daniel Pereira[a], Mariana Sardo[a], Igor Krivtsov[b], Luís Mafra[a*], Marina Ilkaeva[b*]*

[a] Department of Chemistry, CICECO – Aveiro Institute of Materials, University of Aveiro, Campus Universitário de Santiago, 3810-193, Aveiro, Portugal

[b] Department of Chemical and Environmental Engineering, University of Oviedo, 33006 Oviedo, Spain

[c] Institute of Electrochemistry, Ulm University, Albert-Einstein-Allee 47, 89081 Ulm, Germany

*E-mail: ilkaevamarina@uniovi.es (M. Ilkaeva), krivtsovigor@uniovi.es (I. Krivtsov), and lmafra@ua.pt (L. Mafra).

Table of Contents

S1.1 Materials and methods 4

Synthesis of NaPHI 4

Ion-exchange of NaPHI 5

Solid-state nuclear magnetic resonance (ssNMR) spectroscopy 6

Scanning Electron Microscopy (SEM) imaging and Energy Dispersive Spectroscopy (EDS) 6

Scanning Transmission Electron Microscopy (STEM) imaging 6

Thermogravimetric Analysis (TGA) 7

Elemental Analysis (EA) 7

Inductively coupled plasma (ICP) 7

Attenuated Total Reflectance-Fourier Transform Infrared (ATR-FTIR) spectroscopy 7

Powder X-Ray Diffraction (PXRD) 8

Textural properties analysis and manometric CO_2 and N_2 adsorption 8

Thermogravimetric CO_2 and N_2 adsorption studies 8

S1.2 Optimization of synthetic conditions and adsorption studies 10

Table of Supporting Data

Figure S1. **A**: PXRD diffractograms comparing **PCN** with **NaPHI_T** samples and **NaPHI**; **B**: Close comparison between **NaPHI_10**, **NaPHI_15**, **NaPHI_25**, and **PCN** 10

Figure S2. TGA measurements of CO_2 adsorption on **NaPHI** after different pretreatment temperatures. 11

Figure S3. TGA adsorption measurements of **A**: CO_2 and **B**: N_2 uptakes for **PCN**, **NaPHI_T** samples, and **NaPHI** 12

Figure S4. FTIR analyses of **A**: **PCN** and **NaPHI_T** samples, and **B: NaPHI**, and its ion-exchanged analogues 13

Table S1. EA of all NaPHI samples 13

Table S2. EDX of all NaPHI samples 14

Table S3. EA of NaPHI and all its analogues 14

Table S4. EDX of NaPHI and all its analogues 14

Figure S5. TGA analysis of the **NaPHI** batches prepared at different times at 650 ºC. 15

Figure S6. TGA analysis of **NaPHI** and all its ion-exchanged analogues. 16

Figure S7. TGA-MS analysis of **NaPHI** and all its ion-exchanged analogues. 17

Figure S8. SEM images of **A:** NaPHI samples obtained under different synthetic conditions, and **B,C**: ion-exchanged NaPHI analogues. 18

Figure S9. STEM images of **A**: **NaPHI** and **B:** $\mathbf{Rb^+}$**/NaPHI**. 19

Figure S10. Adsorption isotherms of **A:** N_2 at -196 ºC and **B:** CO_2 at 0 ºC of **NaPHI, NaPHI_60**, and **NaPHI_15**. 19

Table S5. NaPHI-based samples' data based on: N_2 isotherms at –196 ºC, and CO_2 isotherms at 0 ºC. 20

Figure S11. A: Fitting of **Zeolite 13X's** N_2 isotherm at –196 ºC to the GCMC model; **B:** the resulting pore size distribution. 20

Figure S12. Fitting of **A: NaPHI, B:** $\mathbf{K^+}$**/NaPHI** and **C:** $\mathbf{H^+}$**/NaPHI** CO_2 isotherm, at 0 ºC, to Dubinin-Stoeckli's model; **D**: the resulting pore size distribution. 21

Figure S13. A: TGA N_2 adsorption measurements and **B:** N_2 adsorption isotherms for **NaPHI** and its ion-exchange analogues. 21

Table S6. Application of IAST equations to CO_2 and N_2 isotherms, at 25 ºC, of NaPHI and its analogues 22

Table S7. Application of Henry's law to CO_2 and N_2 isotherms, at 25 ºC, of NaPHI and its analogues 22

Table S8. Comparison between the selectivity values obtained by applying IAST equations versus Henry's law.. 22

Figure S14. Fitting of **NaPHI**, its ion-exchanged analogues, and **Zeolite 13X's** CO_2 and N_2 isotherms, at 25 ºC, to Dual-site Langmuir and Henry models of adsorption........... 23

Figure S15. Fitting of **NaPHI**, its ion-exchanged analogues, and **Zeolite 13X**'s CO_2 and N_2 isotherms, at 25 ºC, to the Henry model of adsorption. 24

Table S9. Comparison of the CO_2 adsorption capacity values of NaPHI and Zeolite 13X under different temperatures.. 25

Figure S16. CO_2 adsorption capacity from TGA tests for **NaPHI and Zeolite 13X A**: adsorption at different temperatures, and adsorption-desorption cycles at 30 ºC. 25

Figure S17. CO_2 adsorption-desorption recyclability cycles of NaPHI. 26

Figure S18. PXRD patterns of a fresh NaPHI sample against the NaPHI sample after being subjected to 20 cycles of CO_2 adsorption-desorption at 200 ºC. 27

Table S10. Comparison of different adsorbents for CO_2 adsorption at a post-combustion capture temperature range 27

S1 Experimental Section

S1.1 Materials and methods

All chemicals were used as received without further purification. All gases were purchased from Air Liquide. The purity of CO_2 was 99,995% and of N_2 - 99,999%.

Melamine (99%) was provided by Sigma Aldrich. Sodium chloride (≥99.9%) and potassium chloride (98%) were provided by VWR CHEMICALS. Rubidium chloride (99%) and caesium chloride (≥99%) were provided by Thermo Scientific. Hydrochloric acid, [≥37% (PA)] and lithium chloride (99.0%) were provided by Merck. For the dialysis, a 15 kD MWCO dialysis tubing [45 mm Flat-width, 10 meters/roll (33 ft)] from Spectrum Laboratories was used. Molecular sieve, Zeolite 13X, was provided by ThermoFischer.

Synthesis of NaPHI

Sodium poly(heptazine imide) (NaPHI) samples were prepared by an ionothermal approach. For the synthesis of NaPHI a modified procedure originally described by Dontsova et al. was implemented[1]. Melamine (4.0 g, 99%) was ground with NaCl (20.0 g, 99.9%) in a mortar. The resulting powder was transferred into an alumina crucible, covered with a lid, and placed in a pre-heated muffle furnace at 670 ºC, for 25 minutes, if not stated otherwise. This sample is designated as **NaPHI**.

The alumina crucible was then removed from the furnace and allowed to cool down to room temperature (rt). The product was washed with distilled water and centrifuged at 3500 rotations per minute (rpm) for 10 minutes (per cycle). This process was repeated until the powder could no longer be sedimented by centrifugation. After that, the product was dialyzed against 5 L of distilled water (H_2O) over a period of 3 days (water was changed twice a day). The contents of the dialysis membrane were then transferred into a beaker and left to dry in an oven, at 70 ºC, for 1 day, yielding a yellow powder (~ 1.5 g), which was stored at rt until further use. 9 batches in total were synthesized, and then mixed and treated as one sample, based on the similarity of each of the batches' PXRD diffractogram. Other sodium PHI samples were also synthesized at 650 ºC at dwelling times in the muffle furnace of 10, 15, 25, 45, 60, and 90 minutes. These samples are designated as "**NaPHI_t**", where t – is dwelling time in the muffle at 650 ºC (e.g. **NaPHI_90** is the sample prepared at 650 ºC for 90 min).

Polymeric carbon nitride (PCN) was prepared for the sake of comparison by thermal condensation of melamine at 550 ºC for 4 h in a lid-covered alumina crucible.

NaPHI-based samples' synthesis conditions.

Name	Time (min)	Temperature (ºC)
**NaPHI_10**	10	650
**NaPHI_15**	15	
**NaPHI_25**	25	
**NaPHI_45**	45	
**NaPHI_60**	60	
**NaPHI_90**	90	
**NaPHI**	25	670

Ion-exchange of NaPHI

For the exchange of sodium for other metal cations, **NaPHI** (0.5 g) was dispersed in 50 mL of a metal chloride (KCl, RbCl, and CsCl) solution (0.1 M, aq.) or of a hydrochloric acid solution (1.0 M, aq., 50 mL), in H_2O. The suspension was taken to an ultrasound bath for 5 min to allow further particle dispersion. Then, this mixture was left to stir, for 24 h, at rt. The product was washed with distilled water and centrifuged at 3500 rpm for 10 min (per cycle). This process was repeated until the powder could no longer be sedimented by centrifugation. After that, the product was dialyzed against 5 L of H_2O over a period of 3 days (water was changed twice a day). The contents of the dialysis membrane were then transferred into a beaker and left to dry in an oven, at 70 ºC, for 1 day, yielding a yellow powder (~ 0.3 g), which was stored at rt until further use. In this work, the samples originated from ion-exchange with metal chloride or hydrochloric acid solutions will be designated as "**X/NaPHI**", where X is the cation introduced by ion-exchange.

Ion-exchanged analogues of NaPHI

Name	Original sample
**K^+/NaPHI**	NaPHI
**Rb^+/NaPHI**	
**Cs^+/NaPHI**	
**H^+/NaPHI**	

Solid-state nuclear magnetic resonance (ssNMR) spectroscopy

All ^{13}C ssNMR spectra were acquired on a Bruker Avance III 400 spectrometer operating at an external magnetic field (B_0) of 9.4 T, with a ^{13}C Larmor frequency of 100.6 MHz and a ^{1}H Larmor frequency of 400.13 MHz.

All experiments were performed on a double-resonance 4-mm Bruker Magic-Angle-Spinning (MAS) probe at a MAS frequency of 11111 Hz, under rt conditions. Samples were packed into ZrO_2 rotors with Kel-F caps, after a pretreatment in the vacuum line, at 200 ºC, for 2h. ^{13}C chemical shifts are expressed in ppm after referencing with α-glycine (secondary reference, C=O, at 176.5 ppm). The ^{1}H ONE PULSE spectra were acquired using a 90º pulse of 3.25 μs (76.92 kHz). The recycle delay (D_1) was set to 5 s. The number of scans (ns) used to record the spectra was 32. The cross polarization (CP) spectra were acquired based on fulfilling the Hartmann-Hahn condition at the radio frequency (rf) field strengths of 312.6 kHz in the ^{13}C channel and 75.8 kHz in the ^{1}H channel with a contact time (CT) of 3 ms. The D_1 was set to 5 s. The ns used to record the spectra was 16152. The high-power decoupling (HPDEC) spectra were acquired. The D_1 was set to 300 s. The ns used to record the spectra was 736.

Scanning Electron Microscopy (SEM) imaging and Energy Dispersive Spectroscopy (EDS)

SEM imaging was performed using a JEOL-6610LV microscope with incorporated Oxford Instruments EDS microanalysis. The images were taken with magnifications from 550 to 15000 times, at 20 kV. Samples were prepared in powder form, by depositing the samples' powder on black carbon tape followed by Au magnetron spattering, to enhance the material's conductivity. Three different aggregates in total were selected for EDS, per sample analyzed. For each aggregate chosen, three different areas were chosen for EDS. The retrieved EDS data represents an average of the values of all three different areas of three different aggregates analyzed. Results are presented in at.%.

Scanning Transmission Electron Microscopy (STEM) imaging

STEM imaging was performed using a JEOL-2200FS microscope with integrated STEM mode. The images were taken with magnifications from 18k to 600k times, at 200 kV. Samples were prepared by dispersing around 5 mg of the sample powder in analytical

grade ethanol (PA, 99.9% purity). To aid in dispersing the particles, the powder in ethanol was left in the ultrasound bath for 5 minutes. Then, a drop of the resulting dispersion was pipetted onto a carbon-coated copper grid, left to dry, and then analyzed.

Thermogravimetric Analysis (TGA)

TGA was performed using a Mettler-Toledo TGA/SDTA851 instrument coupled to a Pfiffer-Vacuum Thermostar GSD301T mass spectrometer. For this equipment, around 20-30 mg of sample were required. The sample mass was introduced in a crucible and then analyzed. Taring of the crucible was conducted before every measurement. The samples were subjected to thermal degradation, under He atmosphere, raising the temperature from 25 ºC to 1000 ºC, at a rate of 5 ºC/min. Throughout the duration of this treatment, the produced gases were analyzed by mass spectrometry by retrieving data related to the following masses: H_2O (m/z = 18), NO_2 (m/z= 46), NO (m/z = 30), CO_2 (m/z =44), N_2 (m/z = 28, 14), O_2 (m/z = 32)

Elemental Analysis (EA)

CHN elemental analyses were performed with a Vario Macro CNHS Analyser. For these analyses, 45-50 mg of sample were required. The combustion furnace's temperature was at 1150 °C. The detection method was thermal conductivity for all elements (C, H, and N).

Inductively coupled plasma (ICP)

ICP analyses were conducted in a 7900x series ICP-MS by Agilent Technologies. For these analyses, 20-30 mg of sample were required. The sample (solid phase) was digested in a nitric acid solution (liquid phase), thus extracting the cations into the liquid phase. This liquid phase was analyzed, and the acquired data was normalized to the sample mass dispersed in the solution.

Attenuated Total Reflectance-Fourier Transform Infrared (ATR-FTIR) spectroscopy

ATR-FTIR spectroscopy was conducted in a FTIR Bruker Tensor 27 instrument with a Golden Gate ATR. Around 5 mg of sample were required. This sample mass was

introduced over the crystal of the equipment, pressed down and locked in place. FTIR spectra were recorded in transmittance mode, 256 scans were taken in the range of wavenumbers 350-4000 cm^{-1}, with a resolution of 1 cm^{-1}.

Powder X-Ray Diffraction (PXRD)

PXRD was recorded using a Malvern Panalytical Empyrean diffractometer with Cu-Kα radiation. The samples were scanned in 0.02 ° 2θ steps with a count time of 90 seconds per step.

Textural properties analysis and manometric CO_2 and N_2 adsorption

A Microtrac Belsorp MAX II HP was employed to acquire adsorption-desorption isotherms of N_2 at - 196 ºC and 25 ºC, and CO_2 at 0 ºC and 25 ºC. Before said measurements, the samples were degassed under vacuum, at 200 ºC, for 2 hours, using a 5 °C/min heating ramp. Additionally, an in-situ pre-treatment was conducted at the analysis port of the instrument, at 120 ºC, for 60 min. The Brunauer, Emmett and Teller surface area (S_{BET}) and the total pore volume (V_{total}) of the samples were calculated using the N_2 adsorption-desorption isotherms at –196 ºC. Total pore volume was estimated using the quantity adsorbed at a relative pressure (p/p_0) of 0.99. The model used to calculate the pore-size distribution (PSD) from the isotherms of N_2 at –196 ºC was grand-canonical Monte Carlo (GCMC), using the BELMaster software. Tikhonov regularization was applied to all retrieved data.

The Dubinin-Stoeckli model was applied to retrieve the PSD and microporous volume (V_{micro}) of PHI-based materials, such as NaPHI from CO_2 adsorption isotherms at 0 ºC.

Ideal Adsorbed Solution Theory (IAST) selectivities were calculated by applying IAST equations for N_2 and CO_2 isotherms, at 25 ºC, using GraphIAST package, developed by Dautzenberg et al[2].

Thermogravimetric CO_2 and N_2 adsorption studies

A TGA 55 from TA Instruments was used for these studies. The sample mass of 20–30 mg was introduced into a crucible, spread on the bottom of the crucible, and then subjected to the analysis. Taring of the crucible was conducted before every

measurement. N_2 was used as the balance/protective gas. Samples were initially degassed by raising the temperature from rt to 200 ºC, if not stated otherwise, at a rate of 5 ºC/min in a flow of N_2. Then, they were held at this temperature for 2 h. Later, the desired gas would be introduced as the temperature was decreasing from 200 ºC to 30 ºC, at a rate of 5 ºC/min (30 ºC is the adsorption temperature used, unless stated otherwise). For CO_2, the gas would be introduced at a flow rate of 40 mL/min. For N_2, the gas would be introduced at a flow rate of 60 mL/min. The following step would be equilibration of the temperature at 30 ºC and, lastly, holding the samples at this temperature for 2 h. Such non-isothermal conditions of performing the adsorption studies were chosen, to reduce the impact of the purge-gas adsorption on the measurement. For the experiments on **Figure 6B** and **Figure S16B,** the pre-treatment temperature was 200 ºC, before the first cycle of adsorption, and then it decreased to 100 ºC for the following cycles. The time of CO_2 adsorption was 10 min, and the regeneration time was 20 min.

S1.2 Optimization of synthetic conditions and adsorption studies

Varying such parameters as time and temperature of the synthesis allowed us to produce a series of samples based on polymeric carbon nitride (PCN). The diffraction patterns for the synthesized materials are depicted in **Figure S1**. It is seen that the **NaPHI_10** sample did not develop the expected diffraction patterns of NaPHI-based framework and show a rather similar PXRD profile to that of the **PCN**'s[3]. Whereas, at longer thermal treatment times, the ($\bar{1}10$) reflection appears, which can be assigned to the interlayer stacking in NaPHIs[4]. This suggests that for effective alkali metal cation incorporation to occur, longer dwelling times of synthesis are required. Reflections at 8.2° ($\bar{1}10$) and at 14.3° ($\bar{1}20$) correspond to the expected intralayer order and eclipsed conformation of the PHI sheets, respectively[1]. All reflections observed on the registered PXRD patterns are indexed as reported by Lotsch et al[4]. One can also observe that the extension of the dwelling time led to the increase of the relative intensity of the characteristic reflections of the PHI structure. The same effect was achieved by increasing the temperature of the treatment from 650 °C to 670 °C, while maintaining the time of the synthesis of 25 min.

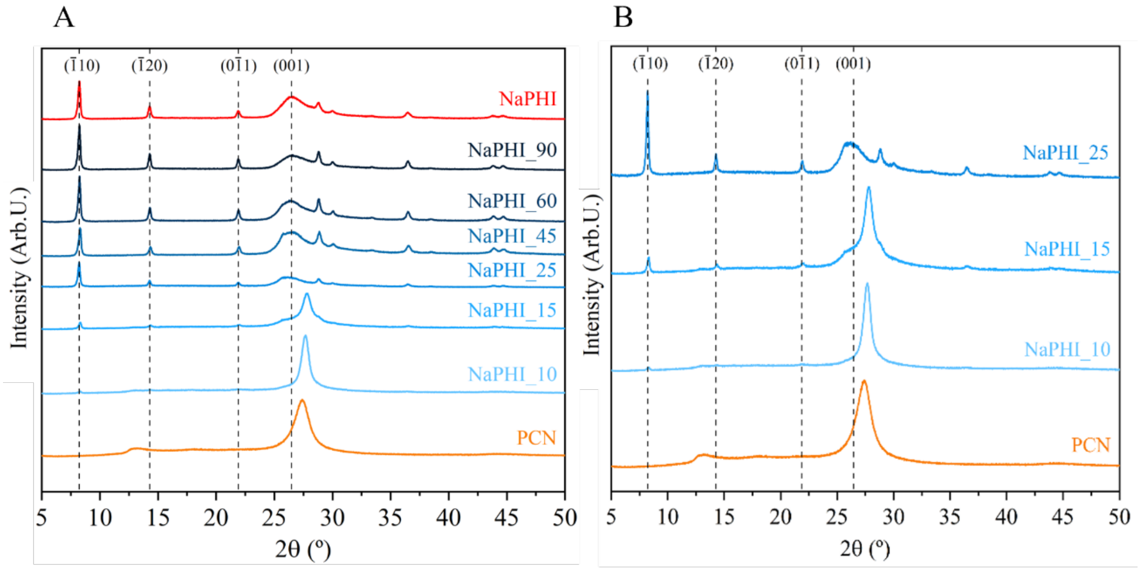

Figure S1. A: PXRD diffractograms comparing **PCN** with **NaPHI_T** (synthesized at 650 °C) samples (where T is the dwell time in minutes) and **NaPHI** (synthesized at 670 °C, for 25 minutes); **B**: Close comparison between **NaPHI_10**, **NaPHI_15**, **NaPHI_25**, and **PCN**.

To test gas adsorption performance of the synthesized materials, CO_2 and N_2 uptakes were determined using thermogravimetric (TGA) technique (**Figure S2,3**). After the optimization of the synthesis conditions, the next step is to optimize the conditions at

which the adsorption measurements are performed. Therefore, we screened a range of pre-treatment temperatures applying the thermal treatment of (i) 100 ºC, (ii) 200 ºC, (iii) 300 ºC, and (iv) 400 ºC (**Figure S2**). It was found that by applying 200 ºC for the desorption of gases from the adsorbent is the optimal condition for the pre-treatment. It allows **NaPHI** to achieve the adsorption capacity towards CO_2 of ca. 3 mmol/g, which is superior to that observed for the pre-treatment temperatures of 300 ºC and 100 ºC yielding 2.5 and 2.0 mmol/g, respectively. Apparently, the high temperatures of pre-treatment result in the loss of adsorption capacity as it is evident from the test performed using 400 ºC for the pre-treatment (**Figure S2**). Perhaps, this indicates the occurrence of a structural transformation of **NaPHI** caused by the thermal treatment. Achieving a complete removal of adsorbed gases and water at a relatively low temperature would increase the cost-effectiveness of our system as less energy would be required to regenerate the adsorbent.

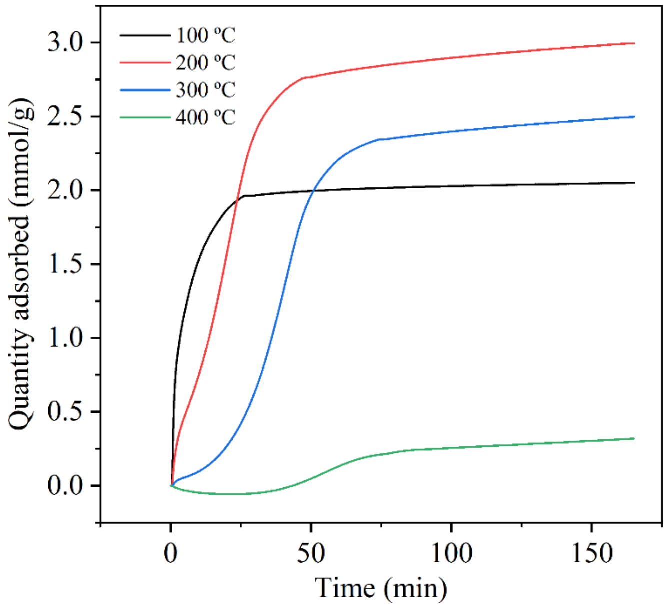

Figure S2. TGA measurements of CO_2 adsorption on **NaPHI** after different pretreatment temperatures.

Therefore, considering the results obtained (**Figure S2**), all the TGA adsorption measurements and manometric adsorption studies performed henceforth were carried out using 200 ºC as the pre-treatment temperature.

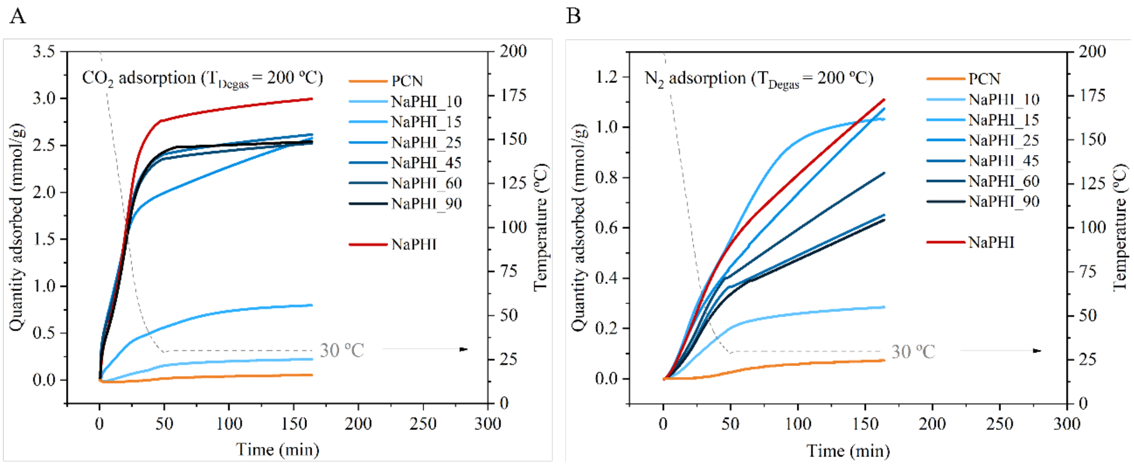

Figure S3. TGA adsorption measurements of **A**: CO_2 and **B**: N_2 uptakes for **PCN**, **NaPHI_T** (synthesized at 650 ºC) samples (where T is the dwell time in minutes), and **NaPHI** (synthesized at 670 ºC, for 25 minutes).

When comparing **NaPHI** sample to the other **NaPHI**-based samples, we observe several advantages to **NaPHI (Figure S3).** Although its synthesis temperature is only slightly higher (670 ºC vs 650 ºC), it is more time-efficient than the best performing sample of the 650 ºC group (25 min vs 45 min). Moreover, **NaPHI** was able to outperform the **NaPHI_45** in terms of (i) adsorption capacity (~ 3.0 mmol/g, at 200 ºC, vs ~ 2.6 mmol/g) (**Figure S3**), and (ii) the synthesis yield is ~ 1.4 g against 1.1 g for **NaPHI_45**, thus making it more practical for scaling-up. Hence, the **NaPHI** sample was chosen to conduct the remaining experiments/measurements presented.

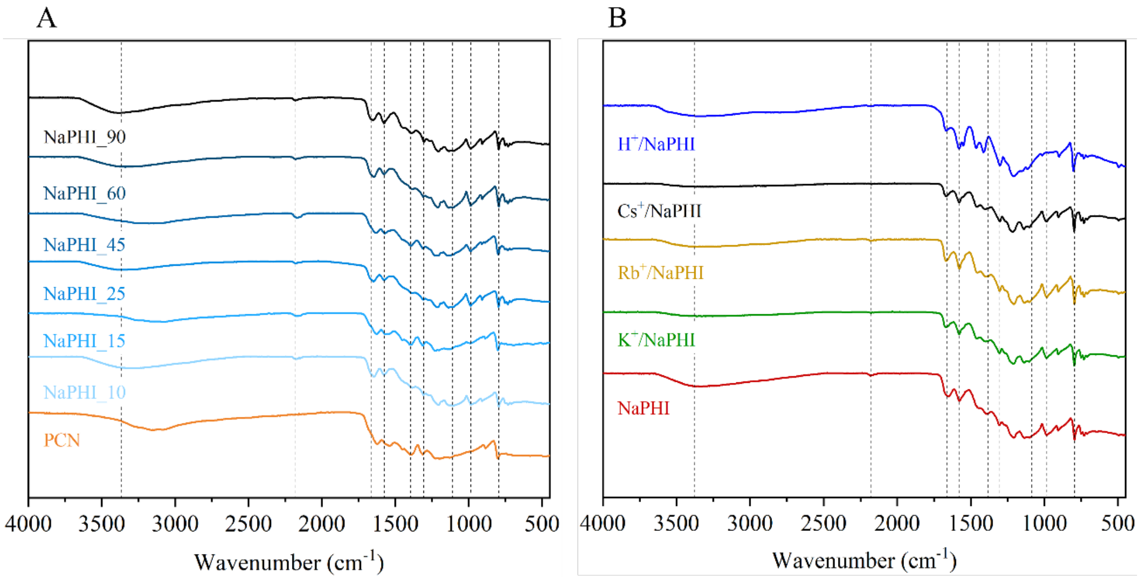

Figure S4. FTIR analyses of **A**: **PCN** and **NaPHI_T** (synthesized at 650 ºC) samples (where T is the dwell time in minutes), and **B**: **NaPHI** (synthesized at 670 ºC, for 25 minutes), and its ion-exchanged analogues.

EA and EDX analyses (**Table S1-S4**) were performed with **NaPHI** and all its analogues to assess the contents of C and N that may reflect the presence of uncondensed amino-groups.

Table S1. EA performed on all samples synthesized at different dwelling times under 650 ºC or 670 ºC (Results are presented in at.%).

Samples	%N	%C	%H	C/N ratio
**NaPHI_10**	58.64	32.91	1.76	0.56
**NaPHI_15**	48.75	28.89	2.41	0.59
**NaPHI_25**	42.71	26.79	2.59	0.63
**NaPHI_45**	43.62	26.46	2.65	0.61
**NaPHI_60**	40.24	26.72	2.38	0.66
**NaPHI_90**	43.75	27.12	2.63	0.62
**NaPHI**	42.06	27.04	2.65	0.64

Table S2. EDX performed on all samples synthesized at different dwelling times under 650 ºC or 670 ºC (Results are presented in at.%).

Samples	%N	%C	%O	%Na	C/N ratio
**NaPHI_10**	60.44	33.98	4.25	1.26	0.56
**NaPHI_15**	49.77	37.42	8.90	3.51	0.75
**NaPHI_25**	50.65	35.72	10.39	3.2	0.71
**NaPHI_45**	47.75	38.88	9.88	3.47	0.81
**NaPHI_60**	53.4	35.54	7.44	3.57	0.67
**NaPHI_90**	55.23	33.98	7.98	2.78	0.62
**NaPHI**	57.29	34.15	6.03	2.52	0.60

Table S3. EA performed on **NaPHI** and all its ion-exchanged analogues (Results are presented in at.%).

Samples	%N	%C	%H
**NaPHI**	42.06	27.04	2.65
**K^+/NaPHI**	42.89	27.10	2.45
**Rb^+/NaPHI**	36.28	2466	2.00
**Cs^+/NaPHI**	34.93	23.77	2.06
**H^+/NaPHI**	43.58	28.63	2.99

Table S4. EDX performed on **NaPHI** and all its ion-exchanged analogues - absence of a value signifies that it was below the limit of detection of the equipment (Results are presented in at.%).

Samples	%N	%C	%O	%Na	%X
**NaPHI**	57.29	34.15	6.03	2.52	-
**K^+/NaPHI**	54.03	40.02	4.08	0.10	1.78
**Rb^+/NaPHI**	54.83	38.97	4.88	0.09	1.58
**Cs^+/NaPHI**	59.04	36.31	3.72	0.26	0.99
**H^+/NaPHI**	56.08	37.78	5.91	-	-

TGA-MS measurements (**Figure S5-S7**) were also employed in this study to evaluate PHI-based materials' thermal stability as well as the products of its thermal degradation process. The analysis demonstrates that gradual development of the NaPHI phase with dwell time under heat treatment is accompanied by the appearance of a mass-loss step centered at around 650 ºC, that likely corresponds to the degradation of the ionic carbon nitride fraction of the material. The samples obtained at shorter synthesis time possess higher number of uncondensed amino-groups, which upon degradation release ammonia and postpone the polymer degradation to higher temperatures.

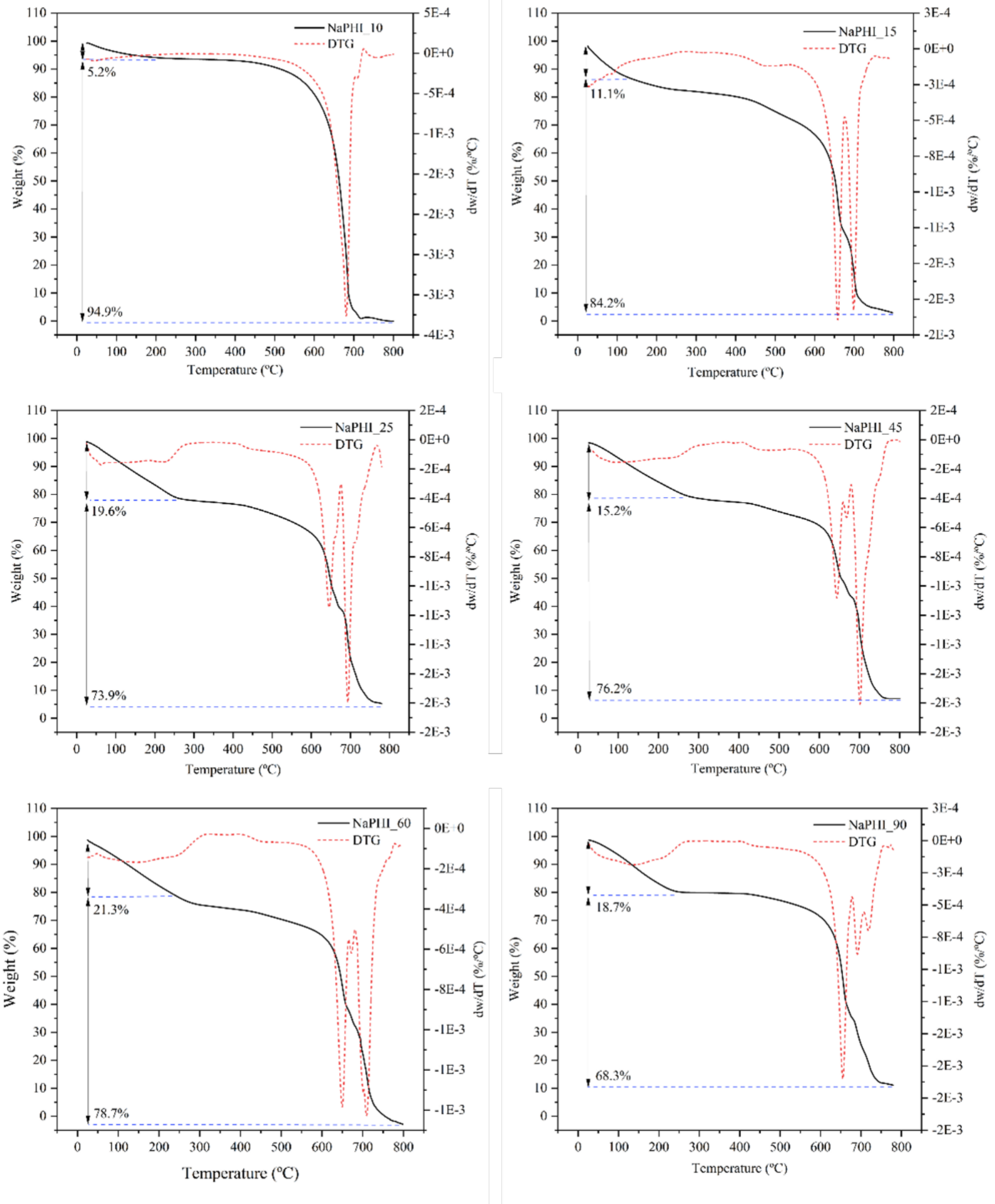

Figure S5. TGA analysis of the **NaPHI** batches prepared at different times at 650 ºC.

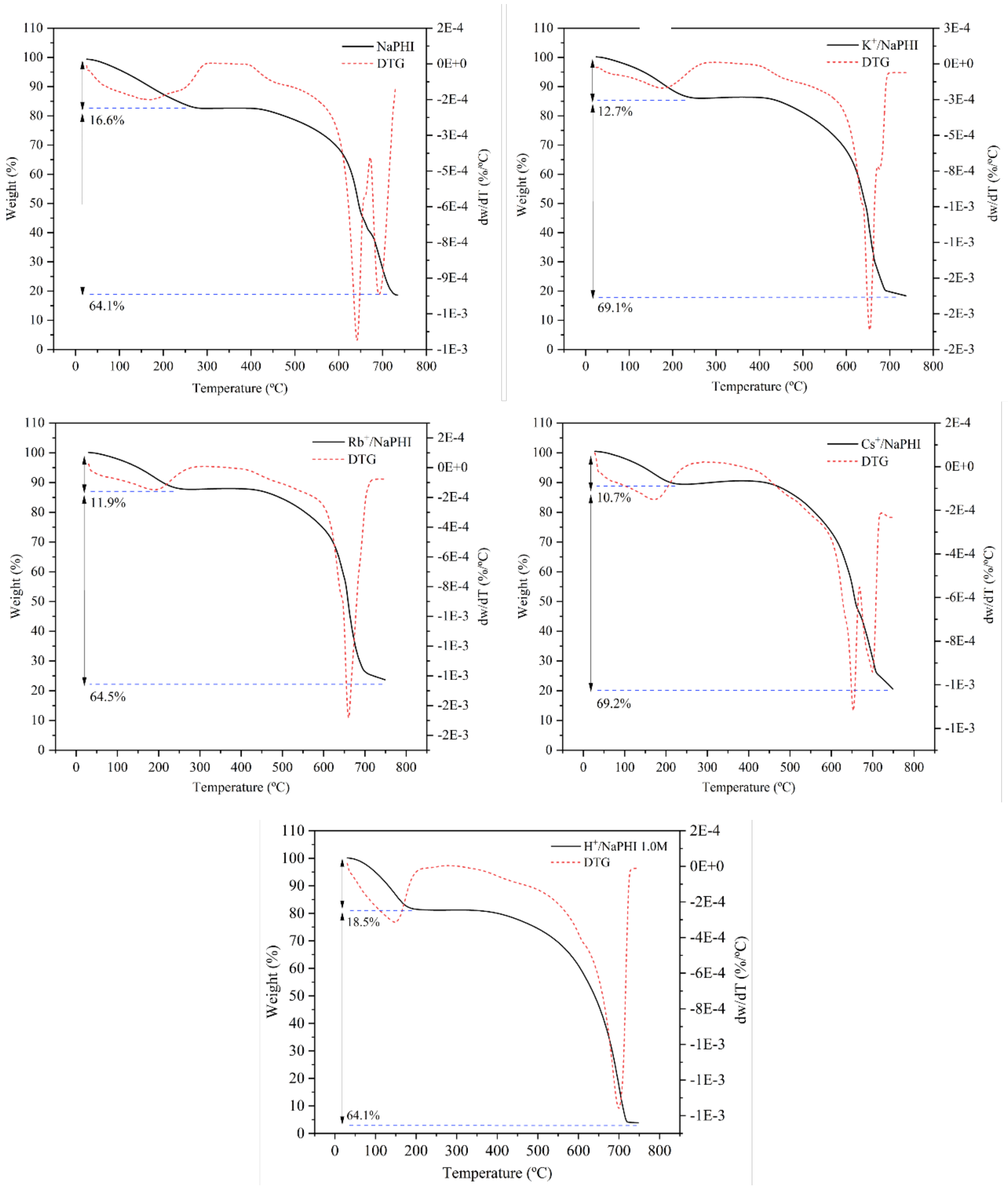

Figure S6. TGA analysis of **NaPHI** and all its ion-exchanged analogues.

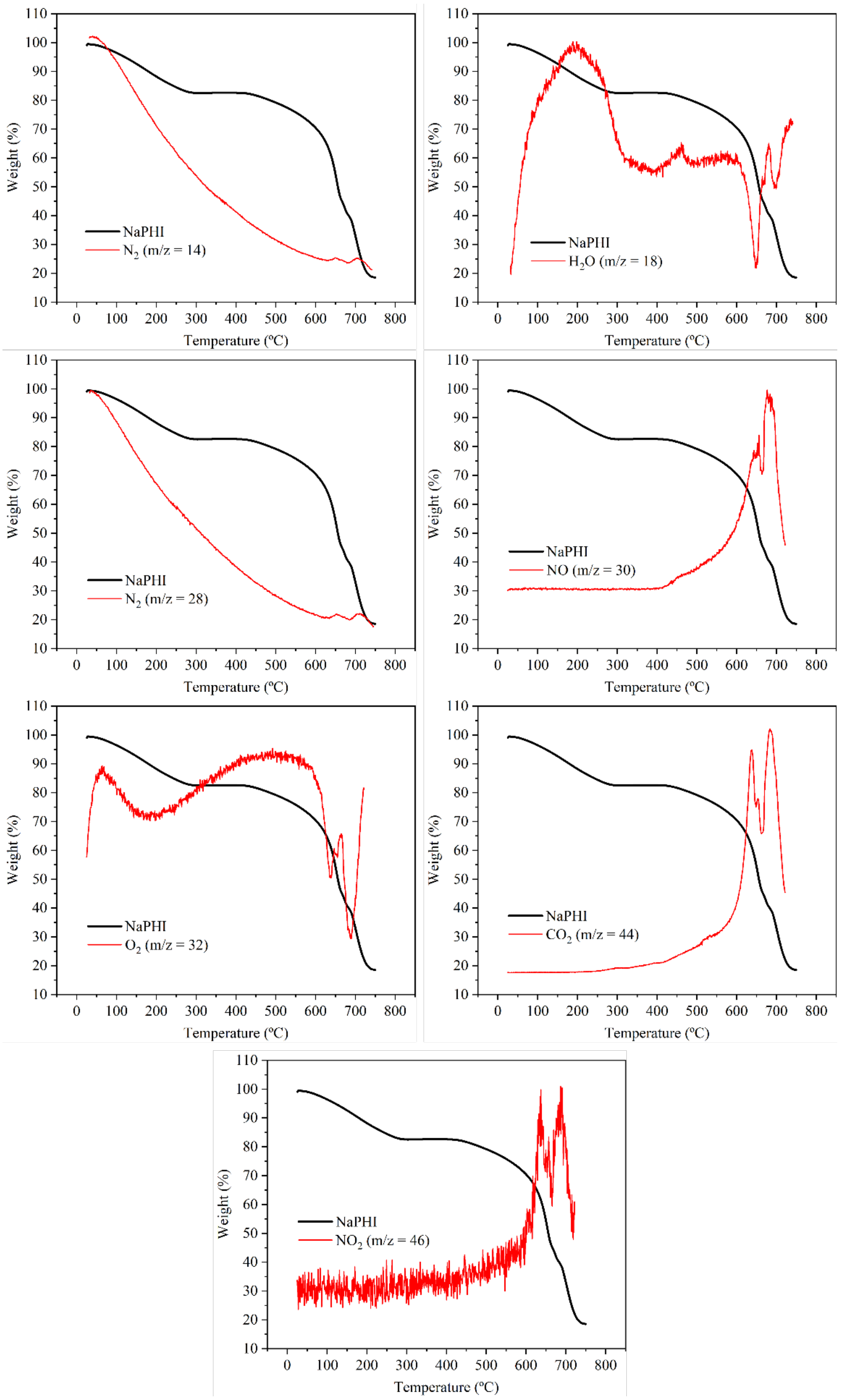

Figure S7. MS signals of the **NaPHI** sample.

SEM imaging was performed to observe how the particles of PHI-based materials change as a function of synthetic conditions, but also to study the effect of ion-exchange on morphology (**Figure S8**). Apparently, the aggregation of the PHI particles is arbitrary, and it yields no defined morphology that could be probed by SEM irrespective of the synthetic conditions and ion-exchange.

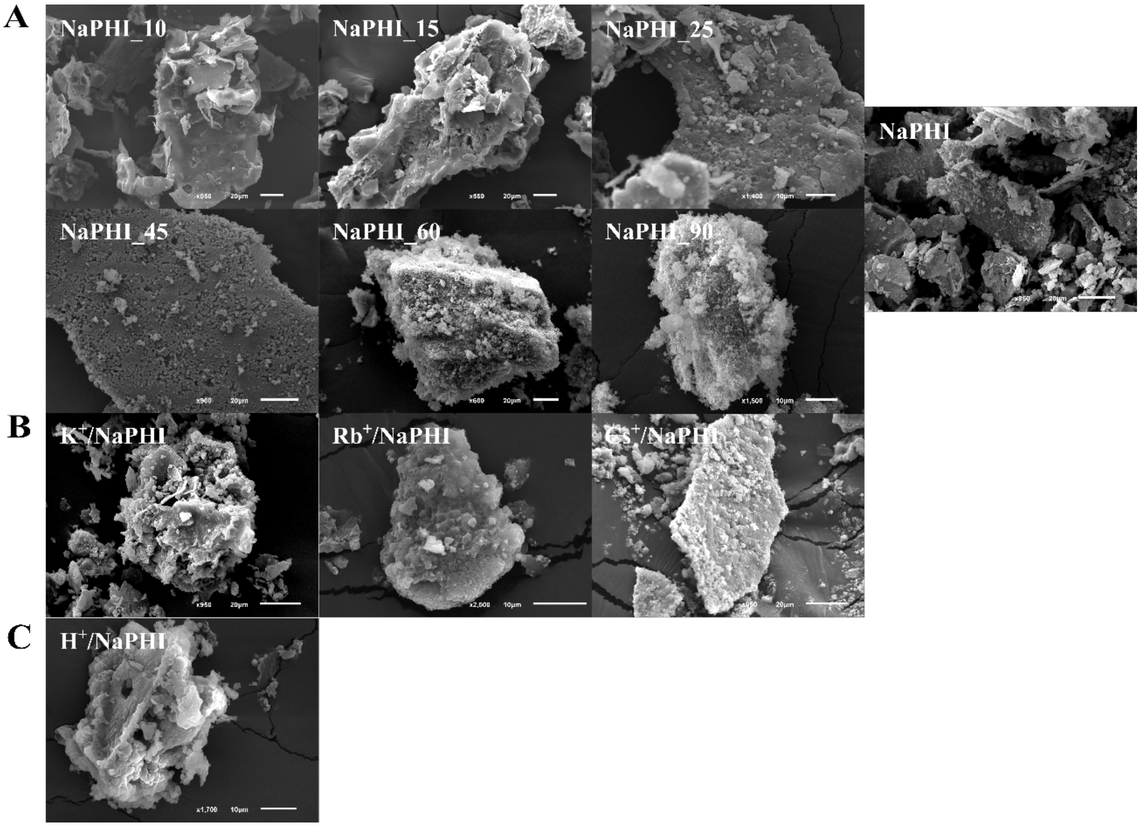

Figure S8. SEM images of **A**: NaPHI samples obtained under different synthetic conditions, and **B,C**: ion-exchanged NaPHI analogues.

STEM, on the other hand, could capture the crystalline particles of the **NaPHI** sample, which is evident by the appearance of the lattice fringes (**Figure S9A)**. Nonetheless, achieving higher magnifications was challenging due to instability of the material under irradiation due to the high energy of the electron beam.

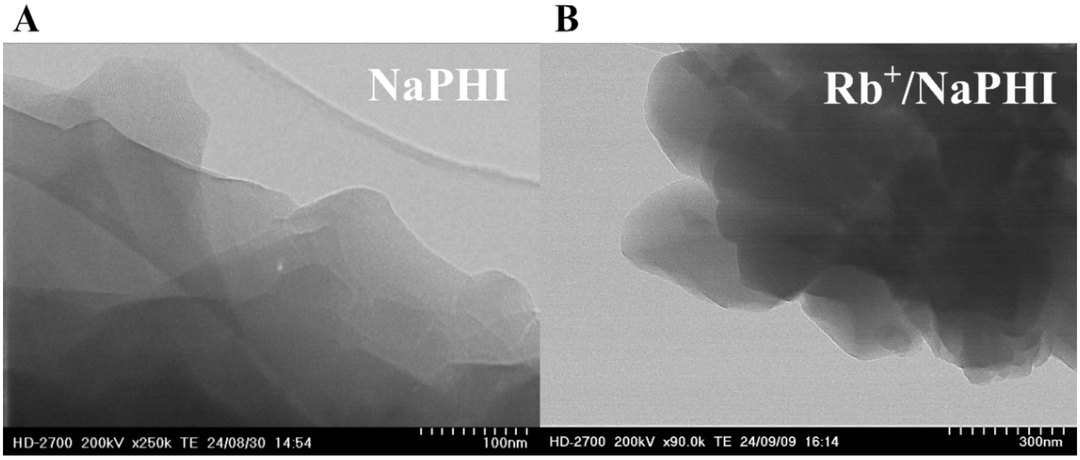

Figure S9. STEM images of **A**: **NaPHI** and **B**: **Rb$^+$/NaPHI**.

N_2 adsorption-desorption isotherms at –196 °C reveal that textural properties drastically change when the dwelling time under heat treatment is increased (**Figure S10**, **Table S5**). An evident increase in the surface area, total pore volume, and CO_2 adsorption capacity is observed when comparing the samples obtained at two dwelling times under the same temperature. The increase of BET specific surface area and the pore volume observed for the **NaPHI_60** sample is likely to be due to exfoliation of the poly(heptazine) sheets rather than to an increase in porosity of the material. It is also evident that such an exfoliation could have resulted in the formation of voids where N_2 condensation at high partial pressures is taking place (**Figure S10A**). This improvement of textural characteristics, however, does not translate into better CO_2 capture performance (**Figure S10B**).

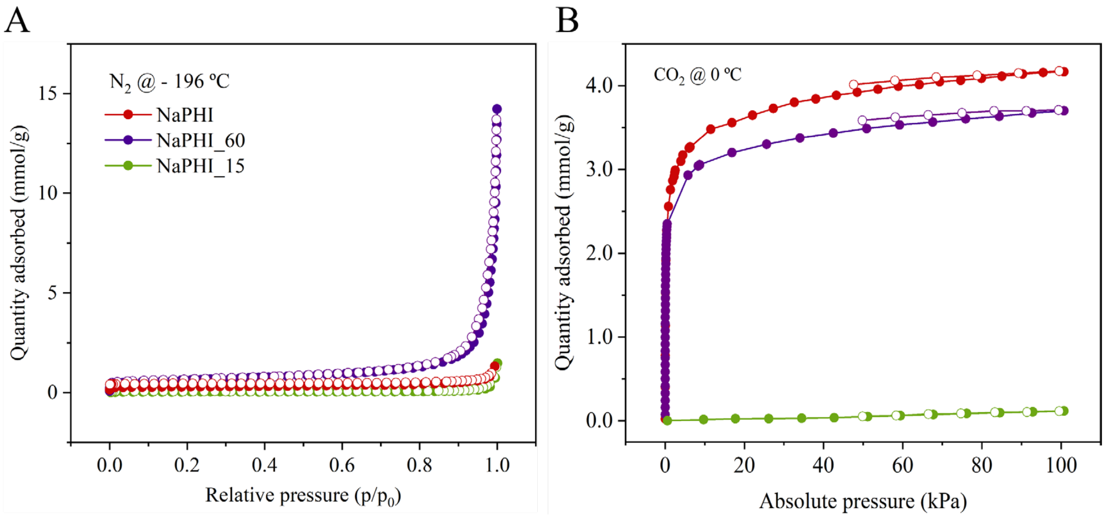

Figure S10. Adsorption isotherms of **A:** N_2 at –196 °C and **B:** CO_2 at 0 °C of **NaPHI**, **NaPHI_60**, and **NaPHI_15**.

Table S5. Data retrieved from the N_2 at –196 ºC and CO_2 at 0 ºC adsorption isotherms of **NaPHI**, **NaPHI_60**, and **NaPHI_15**.

Sample	S_{BET}[a] (m^2/g)	V_{Total}[a] (cm^3/g)	CO_2 0 ºC (mmol/g)	V_{micro}[b] (cm^3/g)
**NaPHI_15**	3	0.02	0.12	0.28
**NaPHI_60**	50	0.26	4.13	0.19
**NaPHI***	25	0.04	4.17	0.21

[a]- data retrieved from N_2 adsorption isotherms at –196 ºC.

[b]- data retrieved from CO_2 adsorption isotherms, at 0 ºC. V_{micro} - total micropore volume calculated from the CO_2 adsorption isotherms at 0 ºC using the Dubinin-Stoeckli model.

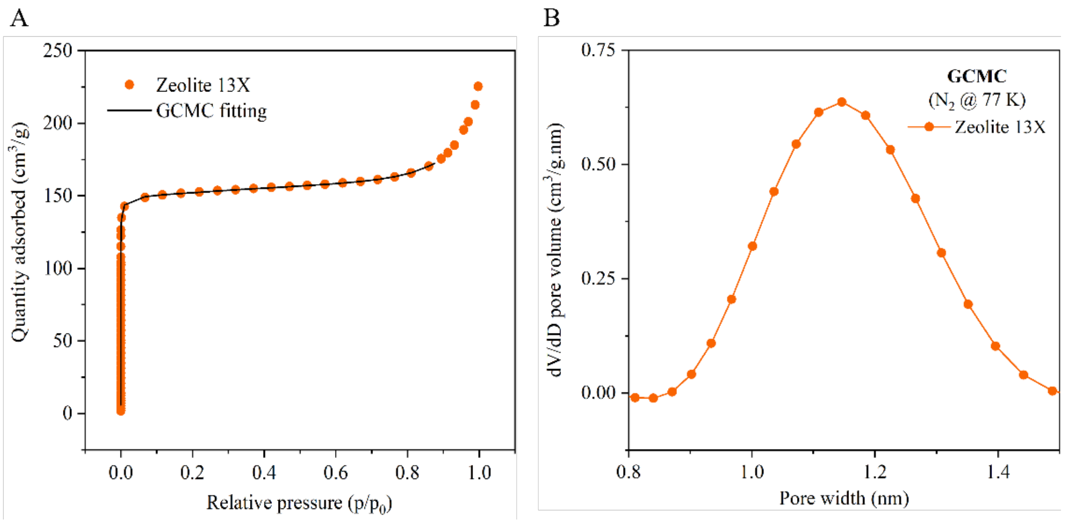

Figure S11. A: Fitting of Zeolite 13X's N_2 isotherm at –196 ºC to the GCMC model; **B:** the resulting pore size distribution.

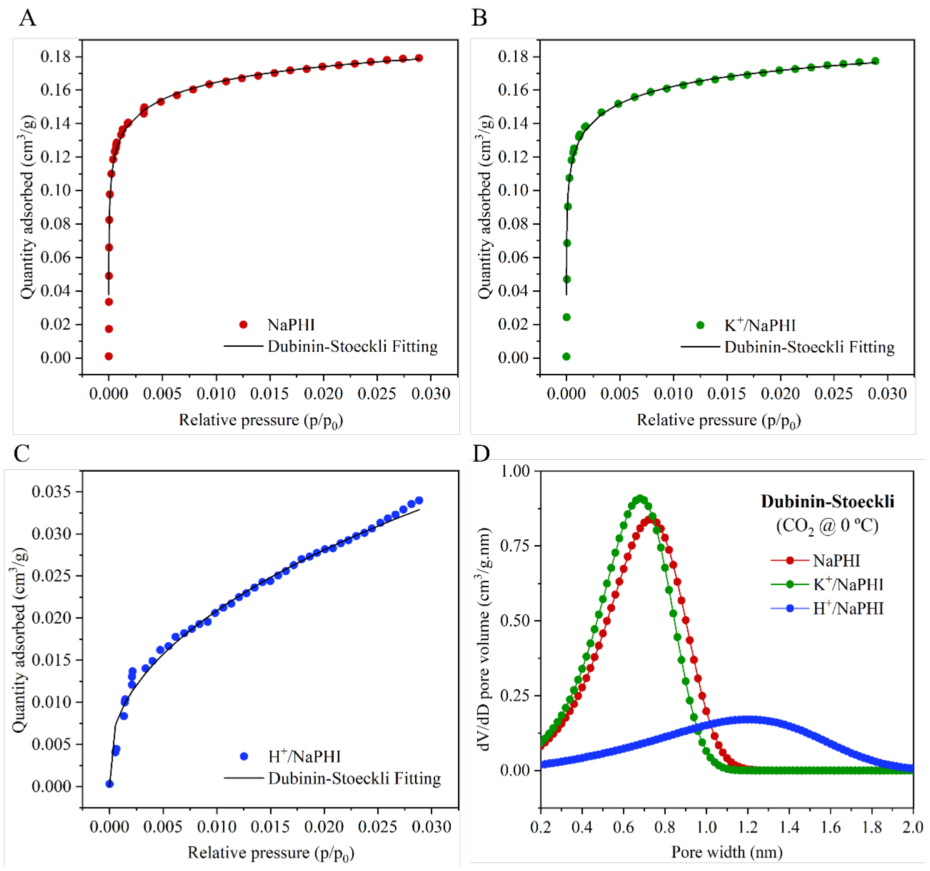

Figure S12. Fitting of **A**: **NaPHI, B: K$^+$/NaPHI** and **C**: **H$^+$/NaPHI** CO_2 isotherm, at 0 °C, to Dubinin-Stoeckli's model; **D**: the resulting pore size distribution.

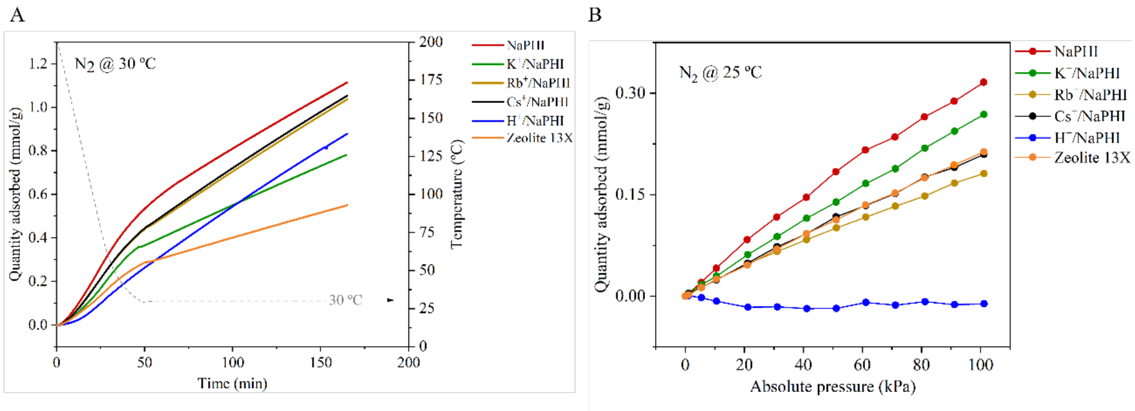

Figure S13. A: TGA N_2 adsorption measurements and **B**: N_2 adsorption isotherms for **NaPHI** and its ion-exchange analogues.

The absence of N_2 uptake by the protonated **H$^+$/NaPHI** counterpart at 25 °C can be explained by the inability of it to efficiently polarize the adsorbate. Moreover, the negative values of adsorption **(Figure S13)** for these samples possibly show that the

material was not fully equilibrated during the pre-treatment stage, and it keeps releasing the trapped gases during the adsorption experiments.

Table S6. Results obtained from applying Dual-Site Langmuir model to CO_2 and N_2 isotherms, at 25 ºC, of **NaPHI** and its ion-exchanged analogues.

**Dual-site Langmuir**	**Samples**				
**Parameters**	**NaPHI**	**K^+/NaPHI**	**Rb^+/NaPHI**	**Cs^+/NaPHI**	**Zeolite 13X**
**K_1 (bar^{-1})**	3.55	0.57	3.13	8.49	1.85
**K_2 (bar^{-1})**	332.73	88.39	72.91	128.33	97.77
**QM_1 (mmol/g)**	1.17	1.49	0.51	1.07	1.02
**QM_2 (mmol/g)**	2.77	3.12	2.36	1.30	3.05
**R^2**	0.9997	0.9997	0.9995	0.9989	0.9997

Table S7. Results obtained from the application of Henry's law to CO_2 and N_2 isotherms, at 25 ºC, of **NaPHI** and its ion-exchanged analogues.

**Henry**	**Samples**				
**Parameters**	**NaPHI**	**K^+/NaPHI**	**Rb^+/NaPHI**	**Cs^+/NaPHI**	**Zeolite 13X**
**K_H (mmol/bar·g) N_2 isotherms**	0.33	0.27	0.19	0.21	0.22
**R^2**	0.9962	0.9994	0.9974	0.9986	0.9996
**K_H (mmol/bar·g) CO_2 isotherms**	776.84	188.91	125.92	110.93	126.65
**R^2**	0.9907	0.9928	0.9809	0.9764	0.9231

Table S8. Comparison between the selectivity values obtained by applying IAST equations versus Henry's law.

**For a 0.04% CO_2 feed**	**Sample**				
**Parameters**	**NaPHI**	**K^+/NaPHI**	**Rb^+/NaPHI**	**Cs^+/NaPHI**	**Zeolite 13X**
**Apparent selectivity**	2354	700	662	528	576
**IAST selectivity**	2487	967	881	745	676

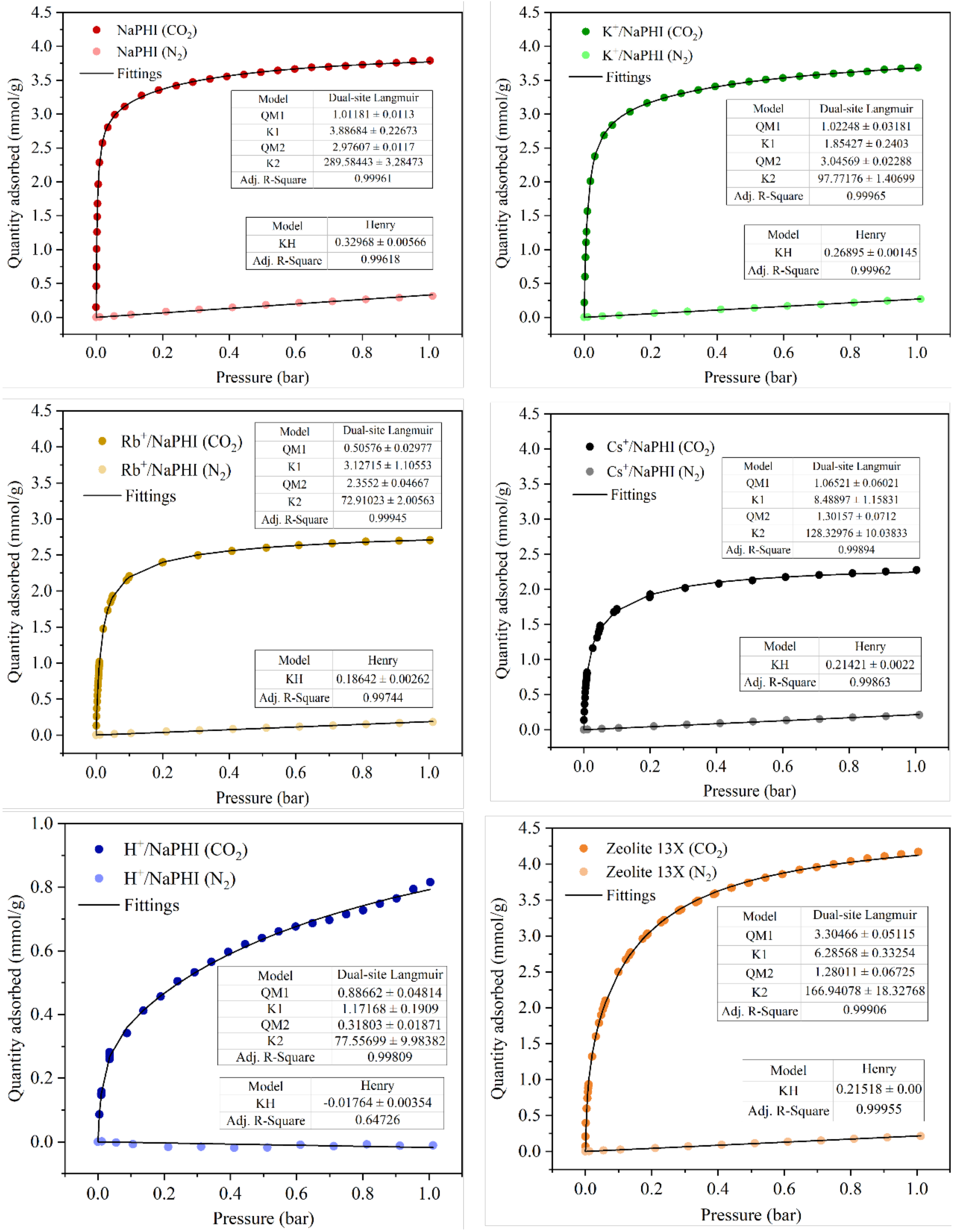

Figure S14. Fitting of **NaPHI**, its ion-exchanged analogues, and **Zeolite 13X's** CO_2 and N_2 isotherms, at 25 °C, to Dual-site Langmuir and Henry models of adsorption.

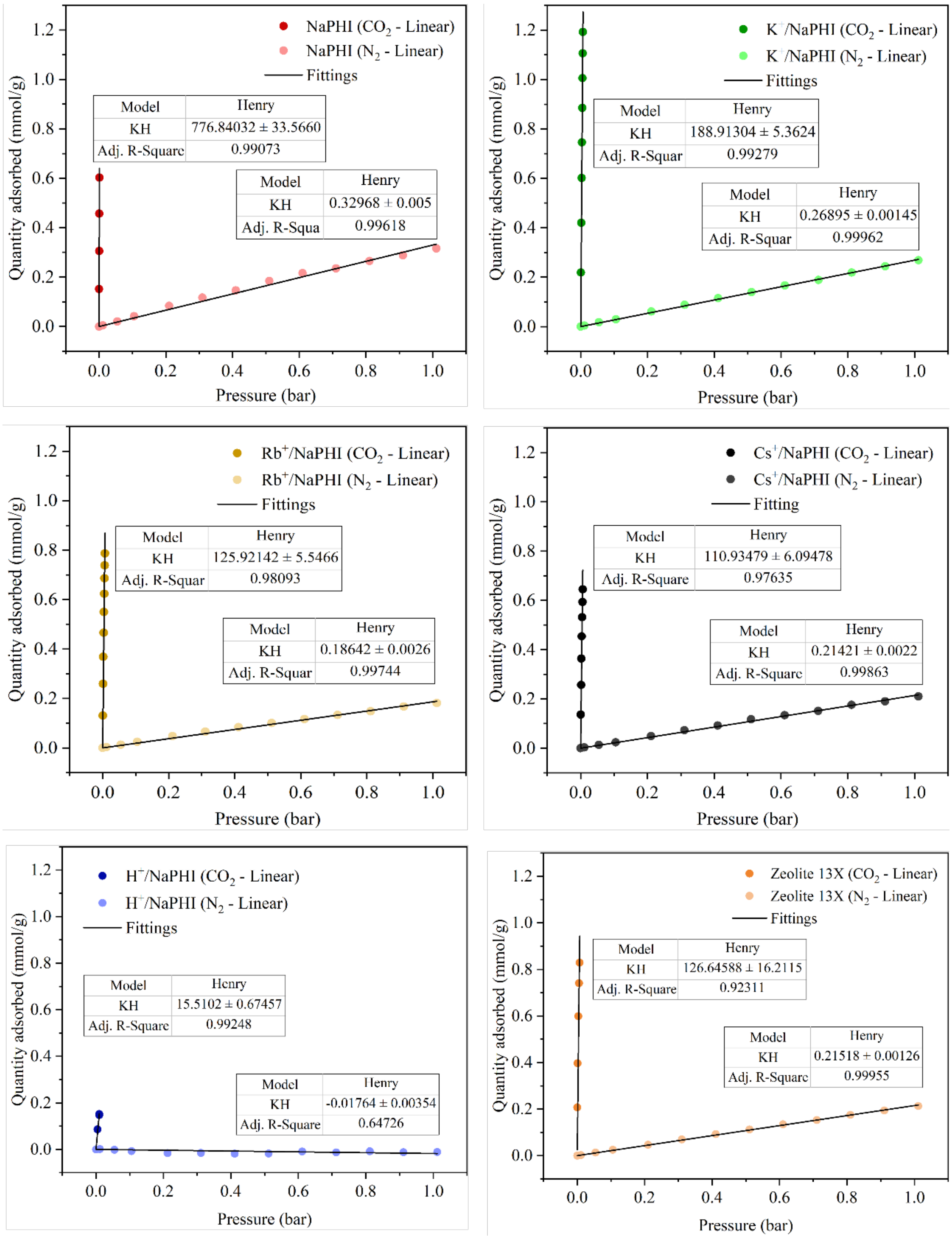

Figure S15. Fitting of **NaPHI**, its ion-exchanged analogues, and **Zeolite 13X**'s CO_2 (linear regions) and N_2 isotherms, at 25 °C, to the Henry model of adsorption.

Table S9. Comparison of the CO_2 adsorption capacity values of **NaPHI** and **Zeolite 13X** under different temperatures (100% CO_2, 1 bar).

Temperature of adsorption (°C)	CO_2 adsorption capacity (mmol/g)		
	**NaPHI**	**Zeolite 13X**	**NaPHI vs Zeolite 13X Adsorption capacity difference (%)**
**30**	2.92	2.99	-2
**50**	2.73	2.26	21
**100**	2.06	1.08	91
**150**	1.17	0.68	72
**200**	0.42	0.30	40

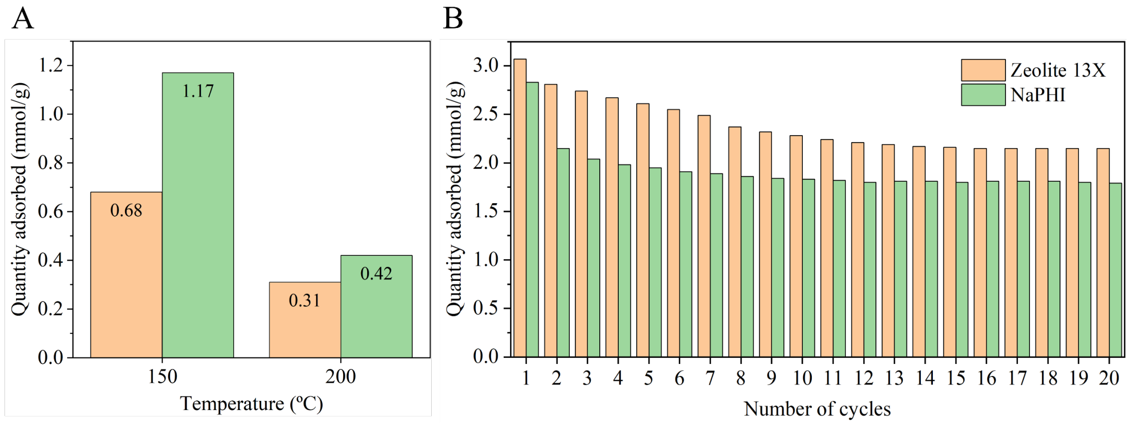

Figure S16. CO_2 adsorption capacity from TGA tests for **NaPHI** and **Zeolite 13X** **A**: adsorption at different temperatures, and **B**: adsorption-desorption cycles at 30 °C. (for **A,** pre-treatment was performed at 200 °C, for 2 hours, under N_2 atmosphere; for **B**, the pre-treatment was 200 °C for cycle 1, then from cycle 2 to cycle 20, it was 100 °C, 20 min for regeneration and 10 min for CO_2 adsorption); CO_2 flow rate for all measurements was 40 mL/min, at 1 bar).

Following the determination of **NaPHI** and **Zeolite 13X's** CO_2 adsorption capacities, the percentage difference between the CO_2 adsorption capacities obtained for the same temperature was calculated (**Table S9**), according to the following formula:

$$\frac{q_{zeolite\ 13X} - q_{NaPHI}}{q_{zeolite\ 13X}} \cdot 100\% \qquad \textbf{(Eq.1)}$$

Where $q_{zeolite\ 13X}$ and q_{NaPHI} are CO_2 adsorption capacities of **Zeolite 13X** and **NaPHI**, respectively.

Through this formula, it is possible to determine how much more CO_2 is **NaPHI** adsorbing in comparison to the **Zeolite 13X**. Results showed that the **NaPHI** starts outperforming the **Zeolite 13X** at temperatures above 30 ºC, reaching its peak at 100 ºC with a 91% difference from the **Zeolite 13X**. Afterwards, this difference decreases with the adsorption temperature increase, however still consistently presenting superior adsorption capacities over the **Zeolite 13X**, regardless.

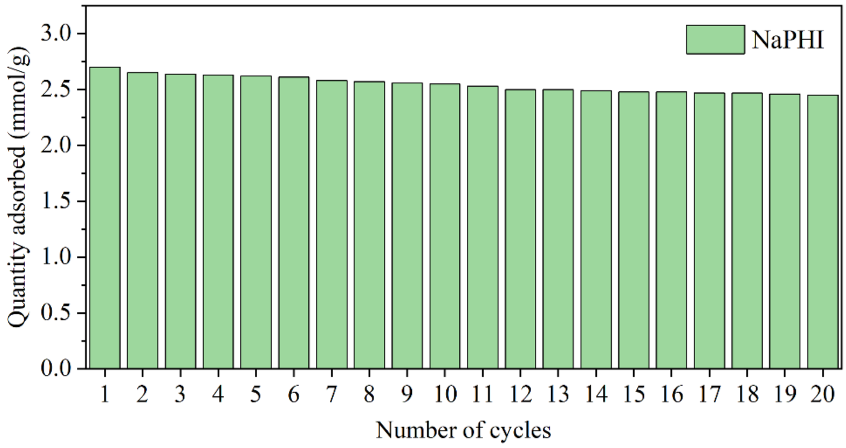

Figure S17. CO_2 adsorption-desorption recyclability cycles of **NaPHI**.

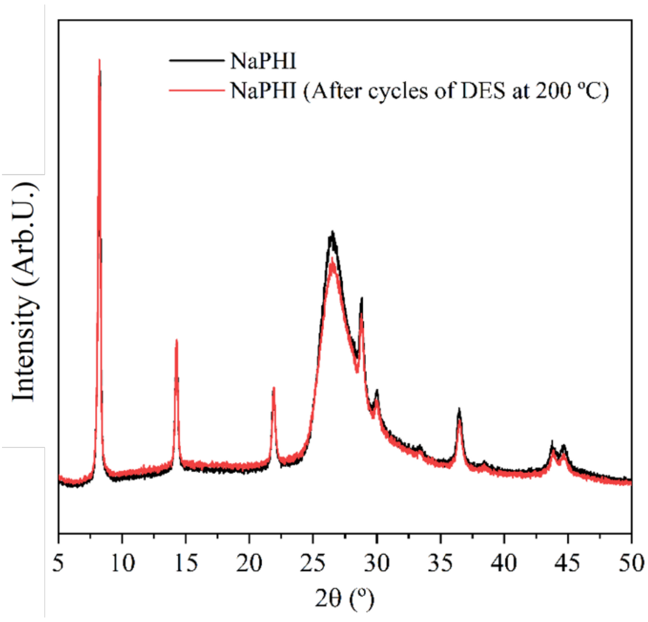

Figure S18. PXRD patterns of a fresh **NaPHI** sample against the **NaPHI** sample after being subjected to 20 cycles of CO_2 adsorption-desorption at 200 °C.

Table S10. Comparison of different adsorbents for CO_2 adsorption at a post-combustion capture temperature range.

**Adsorbent**	**Adsorption temperature (°C)**	**CO_2 adsorption capacity (mmol/g)**	**CO_2 adsorption mechanism**	**Type of measurement**	**Reference**
NaPHI	100	2.06	Physisorption	Gravimetric	This work
Zeolite 13X	100	1.08	Physisorption	Gravimetric	This work
Activated clay	85	0.13	Physisorption	Manometric	5
SBA-PEI	75	2.04	Chemisorption	Manometric	5
Al-MCM-41-100-PEI-50	75	2.75	Chemisorption	Manometric	6
MCM-41	105	0.41	Physisorption	Manometric	7
Norit RBI activated carbon	75	0.91	Physisorption	Manometric	8

References

(1) Chen, Z.; Savateev, A.; Pronkin, S.; Papaefthimiou, V.; Wolff, C.; Willinger, M. G.; Willinger, E.; Neher, D.; Antonietti, M.; Dontsova, D. "The Easier the Better" Preparation of Efficient Photocatalysts—Metastable Poly(Heptazine Imide) Salts. *Adv. Mater.* **2017,** ***29*****, 1700555**. https://doi.org/10.1002/adma.201700555.

(2) Dautzenberg, E.; van Hurne, S.; Smulders, M. M. J.; de Smet, L. C. P. M. GraphIAST: A Graphical User Interface Software for Ideal Adsorption Solution Theory (IAST) Calculations. *Comput. Phys. Commun.* **2022**, *280*, 108494. https://doi.org/10.1016/j.cpc.2022.108494.

(3) Wang, X.; Maeda, K.; Thomas, A.; Takanabe, K.; Xin, G.; Carlsson, J. M.; Domen, K.; Antonietti, M. A Metal-Free Polymeric Photocatalyst for Hydrogen Production from Water under Visible Light. *Nat. Mater.* **2009**, *8*, 76–80. https://doi.org/10.1038/nmat2317.

(4) Schlomberg, H.; Kröger, J.; Savasci, G.; Terban, M. W.; Bette, S.; Moudrakovski, I.; Duppel, V.; Podjaski, F.; Siegel, R.; Senker, J.; Dinnebier, R. E.; Ochsenfeld, C.; Lotsch, B. V. Structural Insights into Poly(Heptazine Imides): A Light-Storing Carbon Nitride Material for Dark Photocatalysis. *Chem. Mater.* **2019**, *31*, 7478–7486. https://doi.org/10.1021/acs.chemmater.9b02199.

(5) Sanz, R.; Calleja, G.; Arencibia, A.; Sanz-Pérez, E. S. CO_2 Adsorption on Branched Polyethyleneimine-Impregnated Mesoporous Silica SBA-15. *Appl. Surf. Sci.* **2010**, *256*, 5323–5328. https://doi.org/10.1016/j.apsusc.2009.12.070.

(6) Xu, X.; Song, C.; Andrésen, J. M.; Miller, B. G.; Scaroni, A. W. Preparation and Characterization of Novel CO_2 "Molecular Basket" Adsorbents Based on Polymer-Modified Mesoporous Molecular Sieve MCM-41. *Microporous Mesoporous Mater.* **2003**, *62*, 29–45. https://doi.org/10.1016/S1387-1811(03)00388-3.

(7) Chaffee, A. L.; Knowles, G. P.; Liang, Z.; Zhang, J.; Xiao, P.; Webley, P. A. CO_2 Capture by Adsorption: Materials and Process Development. *Int. J. Greenhouse Gas Control* **2007**, *1*, 11–18. https://doi.org/10.1016/S1750-5836(07)00031-X.

(8) Van Der Vaart, R.; Huiskes, C.; Bosch, H.; Reith, T. Single and Mixed Gas Adsorption Equilibria of Carbon Dioxide/Methane on Activated Carbon; *Adsorption*; **2000**, *6*, 311–323. https://doi.org/10.1023/A:1026560915422.
